# Hypothalamic remodeling of thyroid hormone signaling during hibernation in the arctic ground squirrel

**DOI:** 10.1038/s42003-022-03431-8

**Published:** 2022-05-23

**Authors:** Helen E. Chmura, Cassandra Duncan, Ben Saer, Jeanette T. Moore, Brian M. Barnes, C. Loren Buck, Helen C. Christian, Andrew S. I. Loudon, Cory T. Williams

**Affiliations:** 1 Institute of Arctic Biology, University of Alaska Fairbanks, 2140 Koyukuk Drive, Fairbanks, AK 99775 USA; 2 Rocky Mountain Research Station, United States Forest Service, 800 E. Beckwith, Missoula, MT 59801 USA; 3 Department of Biology and Wildlife, University of Alaska Fairbanks, 2090 Koyukuk Drive, Fairbanks, AK 99775 USA; 4 Centre for Biological Timing, Faculty of Biology, Medicine and Health, University of Manchester, Manchester, M13 9PT UK; 5 Northern Arizona University, Department of Biological Sciences, 227 Building 21, 617S Beaver, Flagstaff, AZ 86011 USA; 6 Department of Physiology, Anatomy, and Genetics, Le Gros Clark Building, University of Oxford, South Parks Road, Oxford, OX1 3QX UK; 7 Department of Biology, Colorado State University, 1878 Campus Delivery, Fort Collins, CO 80523 USA

**Keywords:** Reproductive biology, Neuroscience

## Abstract

Hibernation involves prolonged intervals of profound metabolic suppression periodically interrupted by brief arousals to euthermy, the function of which is unknown. Annual cycles in mammals are timed by a photoperiodically-regulated thyroid-hormone-dependent mechanism in hypothalamic tanycytes, driven by thyrotropin (TSH) in the pars tuberalis (PT), which regulates local TH-converting deiodinases and triggers remodeling of neuroendocrine pathways. We demonstrate that over the course of hibernation in continuous darkness, arctic ground squirrels (*Urocitellus parryii*) up-regulate the retrograde TSH/Deiodinase/TH pathway, remodel hypothalamic tanycytes, and activate the reproductive axis. Forcing the premature termination of hibernation by warming animals induced hypothalamic deiodinase expression and the accumulation of secretory granules in PT thyrotrophs and pituitary gonadotrophs, but did not further activate the reproductive axis. We suggest that periodic arousals may allow for the transient activation of hypothalamic thyroid hormone signaling, cellular remodeling, and re-programming of brain circuits in preparation for the short Arctic summer.

## Abstract (web summary)

Arctic ground squirrels hibernating in darkness activate the pars tuberalis - hypothalamus thyroid hormone signaling pathway, remodel hypothalamic tanycytes, and activate the reproductive axis.

## Introduction

Hibernation is a phylogenetically ancient^1^ and taxonomically widespread trait in mammals^2^. Characterized by prolonged intervals of metabolic suppression and low body temperature of up to 9 months, it is an adaptive response of animals inhabiting seasonal environments. In small to medium-sized hibernators, metabolism is decreased to 1–2% of basal rates, and body temperature drops to near ambient environment levels as all but essential physiological processes are suppressed^3^. Typically, low temperature torpor is interspersed with brief arousals every few days to weeks, when metabolism and body temperature are restored to euthermic levels; animals subsequently re-enter torpor within 24 h^4,5^. While the function of arousals is debated (many hibernators do not feed or drink during arousal), they are thought to allow for the maintenance of homeostatic processes^4^.

Hibernation requires regulated coordination at behavioral, physiological, and molecular levels prior to initiating dormancy, including energy storage, metabolic adaptations, and inhibition of reproductive function^6,7^. In contrast, aside from transient physiological and neuronal changes associated with arousals^8,9^, little attention has been placed on the potential for seasonal neuroendocrine remodeling across hibernation. In non-hibernating seasonal animals, the latter portion of the non-breeding period is characterized by physiological preparation for reproduction and other seasonal events like migration and molt. For example, seasonal gonadal recrudescence is regulated by neuroendocrine mechanisms that are stimulated by a changing photoperiod weeks to months earlier, leading to extensive physiological, morphological, and behavioral changes^10,11^. Despite early studies finding that initiation of testicular enlargement and transitory increases in gonadotropin and steroid hormones occurs just prior to the end of hibernation in ground squirrels^12,13^, we lack detailed insight as to whether reproductive maturation is preceded by cellular remodeling and other dynamic changes in neuroendocrine circuits across hibernation.

In mammals, the nocturnally-secreted pineal hormone melatonin is critical for transducing seasonal time, and it exhibits marked seasonal decreases in the duration of release from short (SD) to long days (LD). The pituitary pars tuberalis (PT) is rich in melatonin receptors and contains specialized TSH-secreting thyrotroph cells that are responsive to melatonin stimulation^14^. The beta sub-unit of TSH is strongly expressed in response to lengthening photoperiods, and LD-induced TSH acts on receptor fields in adjacent hypothalamic ependymal tanycyte cells, leading to a reciprocal switch in deiodinase enzymes towards LD-associated Dio2 vs Dio3^15^. LD activation of Dio2, in turn, leads to local conversion of circulating thyroxine (T4) to bio-active tri-iodothyronine (T3)^16^, which causes hypothalamic remodeling and neuroendocrine activation in response to LD signals^14,17^. The transcriptional co-activator, *EYA3*, is a critical up-stream regulator of TSHß, and is induced by short-duration nocturnal melatonin signals on long photoperiods^18,19^. Critically, this seasonal timekeeping pathway involving thyrotroph cells in the PT is distinct and separate from the thyroid hormone axis which involves the release of TSH from a distinct population of thyrotrophs in the pars distalis. Core elements of this photoperiodic pathway are conserved across vertebrates^11,14,17,20,21^.

In mammals, changes in T3/T4 in the hypothalamus activate kisspeptin (KISS) neurons in the arcuate nucleus (ARC)^22^, which regulate gonadotropin-releasing hormone (GnRH)^23^, leading to seasonal activation of the gonadal axis^24^. These changes in TH signaling are associated with hypothalamic re-modeling of tanycytes, which have cellular projections extending to the ARC, median eminence (ME), and PT^25^. In a current model emerging from research in birds and mammals, tanycytic end-feet engulf GnRH neuron terminals at the ME during the non-reproductive period and retract during the breeding season to facilitate GnRH release and trigger gonadal development^19,26^. More generally, dynamic tanycyte-neuron interactions are involved in the regulation of metabolic cues that control feeding behavior^27,28^.

In this study, we examine seasonal timekeeping processes during hibernation and under constant darkness in the arctic ground squirrel *(Urocitellus parryii)*. The arctic ground squirrel is endemic to boreal and arctic landscapes and spends as much as 9 months of the year in a hibernating state, during which core body temperatures can decrease to −2.9 °C^29^. For several weeks after completing hibernation under natural conditions, male arctic ground squirrels remain below ground at euthermic body temperatures in constant darkness subsisting on autumnal-stored food caches. This is when most testicular growth and spermatogenesis occurs^30^. In contrast, females emerge above-ground within 3 days of completing hibernation, mate, ovulate, and initiate pregnancy 1–4 days later^31^.

We hypothesized that physiological preparations for spring requires initiation by the central nervous system during hibernation through changes in the tissues and circuits that drive seasonal neuroendocrine function. We show here that during hibernation under constant temperature and darkness there are marked changes in seasonal timekeeping and reproductive pathways within the PT/hypothalamic axis including: upregulation of *TSHß*, structural changes in tanycytic projections to the ME and ARC, and activation of genes implicated in neurogenesis and structural remodeling. Artificial elevation of environmental temperature is known to drive hibernators to terminate hibernation prematurely^32^. Using this protocol, we show that while early resumption of persistent euthermy leads to early reactivation of hypothalamic Dio2 signaling, PT *TSHß* expression is unaffected and gonadal growth is not immediately induced. Collectively, these data suggest that the brains of hibernators exhibit spontaneous seasonal dynamism in the hibernating state, and that seasonal PT/hypothalamic circuitry may be gradually reactivated by a temperature-compensated circannual clock during short arousals across the winter hibernation season.

## Results

### Activation of the PT/tanycyte (TSH/DIO) axis during hibernation

**Fig. 1:**
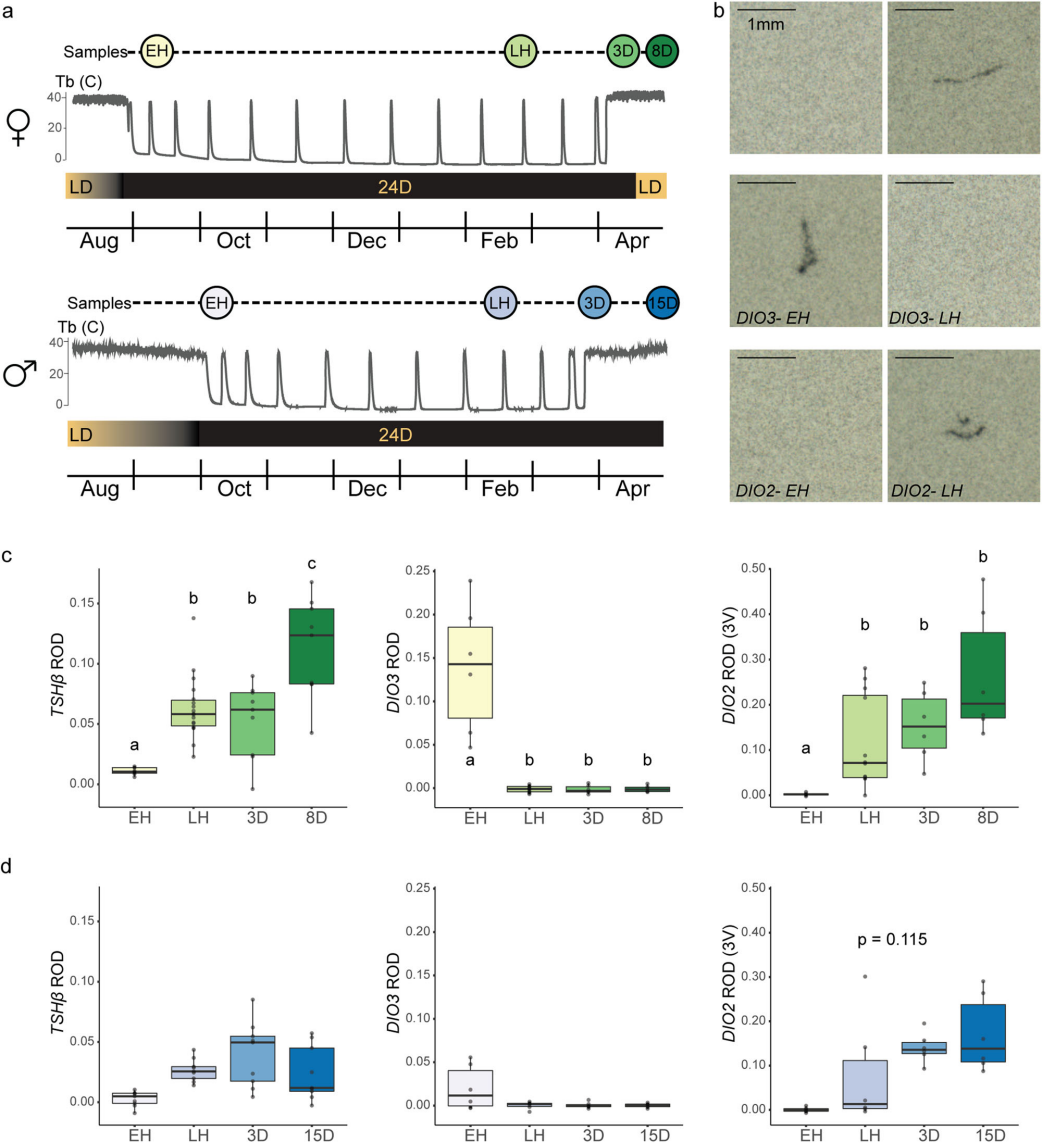
Activation of the PT/tanycyte (TSH/DIO) axis during hibernation. **a** Schematic of repeated cross sectional study in female (top) and male (bottom) arctic ground squirrels. The colored circles represent the tissue collection points (EH: early hibernation; LH: late hibernation; 3D: 3 days post-hibernation, 8D: 8 days post-hibernation; 15D: 15 days post-hibernation); all samples were taken from euthermic animals. The thin gray lines represent body temperatures of arctic ground squirrels before, during, and after hibernation; temperature traces are a schematic. The shaded rectangle indicates the photoperiodic regime experienced by study subjects (LD: long-day; 24D: 24 h dark). Panels **b**–**d** present data depicting mRNA expression of targets associated with retrograde thyroid hormone signaling between the pars tuberalis and hypothalamus as quantified by relative optical density (ROD) of radio-labeled probes during in-situ hybridization. **b** Images of TSHβ, DIO2, and DIO3 staining from EH and LH females. Photos selected to represent highest expression observed in group and scale bars are 1 mm. **c** ROD for females and **d** males for TSHβ in the pars tuberalis and DIO2 and DIO3 along the third ventricle (left to right). Colored shading of boxplots represents sampling groups. Significantly different pairwise comparisons with linear-mixed effects modeling are indicated by lower-case letters (*p* < 0.05). The center line in the box plot represents the median, box boundaries represent the 25th and 75th percentiles, and error bars represent the 25th and 75th percentiles ± 1.5 times the interquartile range. For sample sizes, see methods. See also Supplementary Fig. S1.

We established mixed sex cohorts of wild-caught ground squirrels during late summer in a hibernation facility at the University of Alaska Fairbanks. Animals entered hibernation in fall and were sampled early and late in hibernation, as well as three days post-hibernation, under 2 °C ambient temperatures and continuous darkness (Fig. 1a). Final sampling points occurred either 15 days post-hibernation under constant darkness (males) or 8 days post-hibernation after five days of exposure to a 16L: 8D photoperiod (females); differences in the final sample point reflect natural sexual dimorphism in the annual cycle of arctic ground squirrels^31^. mRNA profiling by in-situ hybridization in the ventral hypothalamus and pituitary PT revealed significant suppression of *TSHβ*, *Dio2*, and elevation of *Dio3* during early hibernation in females (Fig. 1b–d and Supplementary Tables S1, S2). Strikingly, in late hibernation, we observed significant elevation in *TSHβ* and *Dio2* and suppression of *Dio3*. This late-hibernation activation of TH signaling remained largely unchanged in the post-hibernation state (Fig. 1b–d). Seasonal expression patterns in males were similar, although not significant. Expression of *Eya3* was low, but unchanging, in both sexes during hibernation (Supplementary Fig. S1 and Supplementary Tables S1, S2). Collectively, these data reveal that dynamic changes in TSH/TH signaling occur during hibernation, with marked suppression of this axis co-incident with hibernation onset and significant activation later in the hibernation season.

**Fig. 2:**
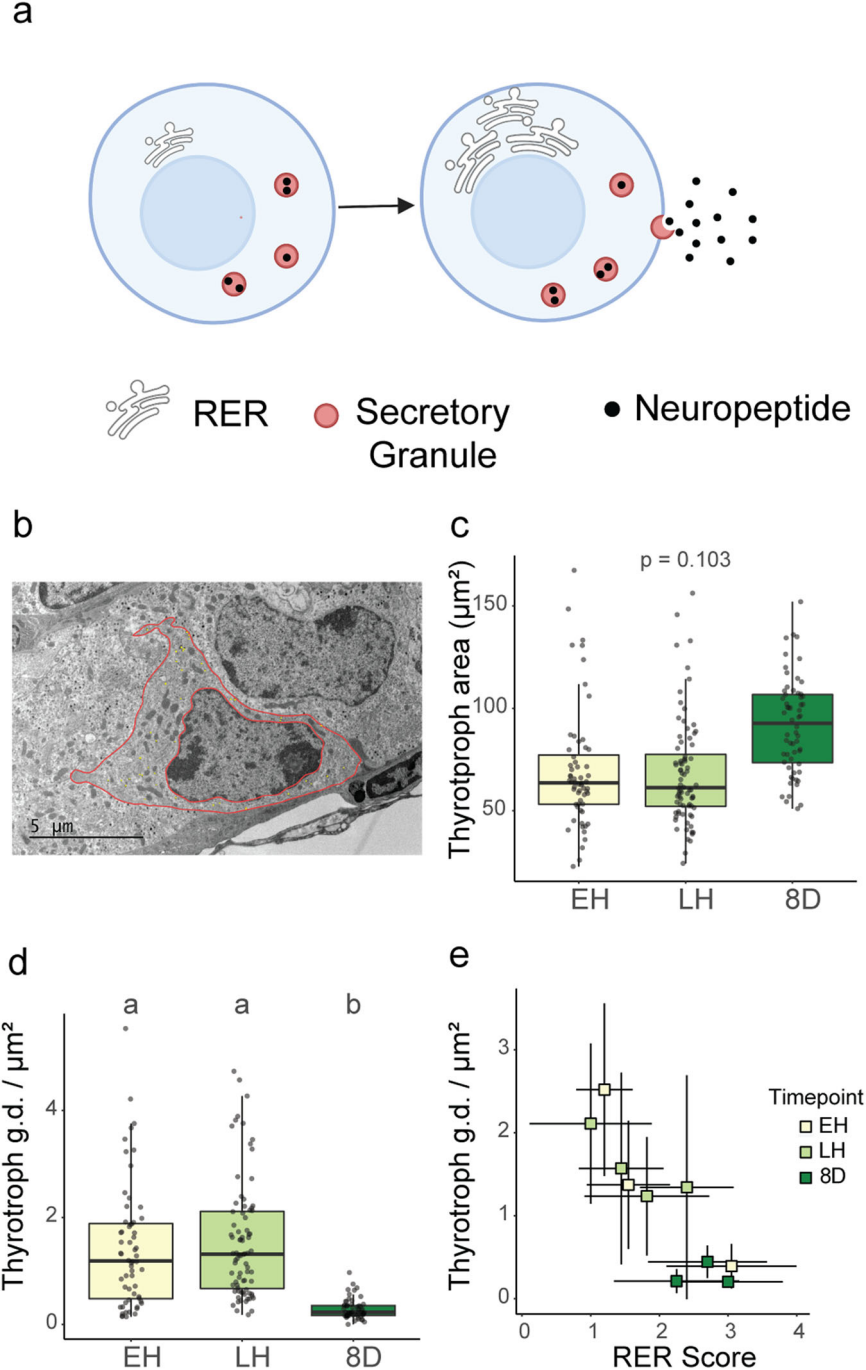
Cellular remodeling of thyrotrophs post-hibernation. **a** Schematic of morphological changes associated with neuropeptide secretion. **b** Electron microscopy (EM) image of thyrotroph cell with outline of the total cytoplasmic and nuclear area shown in false pink coloring. **c** Cross sectional area (μm^2^) and **d** secretory granule density of thyrotroph cells in the pars tuberalis of female arctic ground squirrels. **e** The relationship between individual PT thyrotroph secretory granule density and individual thyrotroph RER (rough endoplasmic reticulum) activity. In panel **e**, data points and error bars represent means ± SD. In all boxplots, colored shading represents sampling groups, the center line in the box plot represents the median, box boundaries represent the 25th and 75th percentiles, error bars represent the 25th and 75th percentiles ± 1.5 times the interquartile range, and lower-case letters indicate significantly different comparisons using linear-mixed effects modeling (*p* < 0.05). For sample sizes, see methods. See also Supplementary Fig. S1.

Prior studies in sheep have demonstrated that photoperiodic activation of the TSH/TH pathway is accompanied by changes in the morphology of PT thyrotrophs^19^. Therefore, we used electron microscopy (EM) to examine whether thyrotrophs were remodeled across hibernation. The size of PT thyrotrophs was similar in early and late hibernation but tended to increase once hibernation was complete (Fig. 2c). Further, PT thyrotrophs showed a significant post-hibernation decrease in secretory granule density (Fig. 2d; Supplementary Table S1), and, as RER abundance increased, granule density decreased, consistent with granule secretion (Fig. 2a, e). Thus, while TSH/TH signaling is activated during hibernation, the amplitude of the signal is likely increased by further post-hibernation remodeling of thyrotrophs.

### Cellular re-modeling of hypothalamic tanycytes during hibernation

**Fig. 3:**
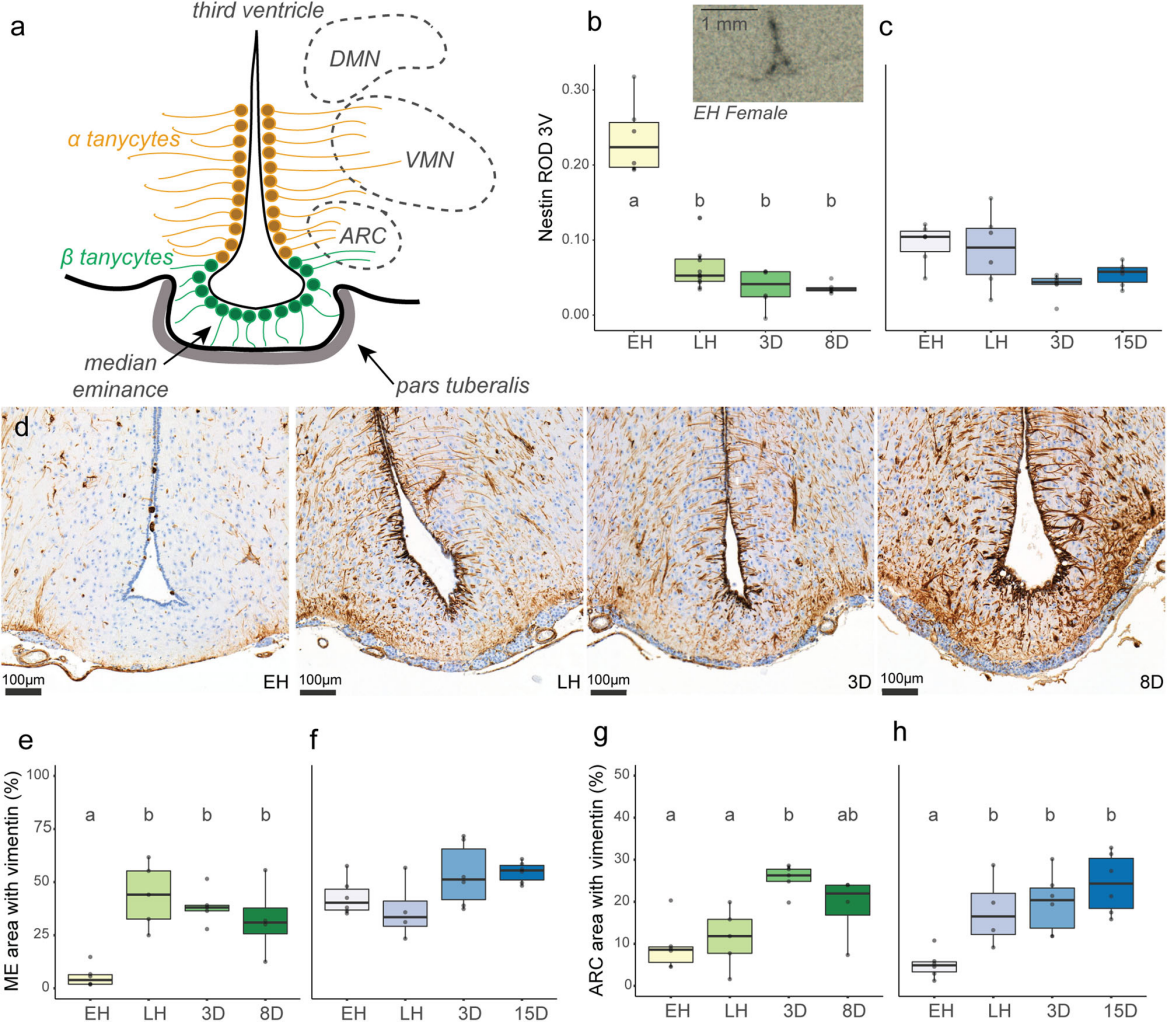
Cellular re-modeling of hypothalamic tanycytes during hibernation. **a** Schematic representing distribution of α (orange) and β (green) tanycytic sub-types along the third ventricle of the hypothalamus. Dashed lines indicate the approximate location of adjacent nuclei (ARC: arcuate nucleus; VMN: ventromedial nucleus; DMN: dorsomedial nucleus). Expression of *nestin* mRNA along the third ventricle of the hypothalamus in female (**b**) and male (**c**) arctic ground squirrels as quantified by relative optical density (ROD) of radio-labeled probes during in-situ hybridization. Inset in panel B shows representative nestin staining in early hibernation females and scale bars are 1 mm. **d** Immunohistochemistry (IHC) of vimentin protein across sampling timepoints in female arctic ground squirrels (one individual per timepoint). Panels **e**–**h** represent quantitative scoring of vimentin in the ME (**e**, **f**) and ARC (**g**, **h**) in females (**e**, **g**) and males (**f**, **h**). For all boxplots, significantly different pairwise comparisons with linear-mixed effects modeling are indicated by lower-case letters (*p* < 0.05). Colored shading represent the different sampling groups, the center line in the box plot represents the median, box boundaries represent the 25th and 75th percentiles, and error bars represent the 25th and 75th percentiles ± 1.5 times the interquartile range. For sample sizes, see “Methods”. See also Supplementary Figs. S1–S4.

We next addressed whether tanycytes, which are key components of the hypothalamic neural network controlling energy balance^27,33^, undergo cellular remodeling during hibernation. Studies in mice suggest that in addition to their role in altering hypothalamic TH availability via changes in deiodinase expression^15^, tanycytes also form a blood–cerebrospinal fluid (CSF) barrier at the ME^34^, integrate and shuttle circulating metabolic signals to hypothalamic neurons^35^, and exhibit neural stem cell properties^36^. We used immunohistochemical staining for Vimentin, an intermediate filament protein and known tanycytic marker^25^ to characterize the tanycyte distribution along the third ventricle and adjacent brain nuclei (Fig. 3a). Across hibernation, hypothalamic Vimentin staining increased dramatically, especially in females. During early hibernation, almost no Vimentin staining was visible in the main tanycyte cell body along the third ventricle and minimal staining was visible in tanycytic projections, whereas by late hibernation, strong Vimentin staining occurred in both tanycytic cell bodies and microfilaments (Fig. 3d, Supplementary Fig. S3, and Supplementary Tables S1, S2). In females, Vimentin staining in the ME increased from early to late hibernation (Fig. 3e, Supplementary Fig. 3, and Supplementary Table S1) and, in the ARC, Vimentin staining increased gradually between early and post-hibernation sampling points (Fig. 3g, Supplementary Fig. S3, and Supplementary Table S1). Males showed a similar, but less striking, pattern with Vimentin staining increasing within the ARC during hibernation (Fig. 3h, Supplementary Fig. S3, and Supplementary Table S2). In contrast, we did not observe changes in immunostaining for monocarboxylate transporter 8 (MCT8), a thyroid hormone transporter expressed in tanycytes (Supplementary Fig. S4 and Supplementary Tables S1, S2). The consistent MCT8 staining of both third ventricle tanycyte cell bodies and their projections throughout adjacent hypothalamic nuclei suggests that tanycytic processes remain prevalent in the ME and ARC throughout hibernation and instead, reductions in Vimentin staining are indicative of structural remodeling. To evaluate this hypothesis, we conducted in-situ hybridization for the intermediate filament nestin (NES), which mediates the disassembly of Vimentin intermediate filaments and has been implicated in both hypothalamic structural remodeling and neurogenesis^36–38^. NES expression showed a marked decrease between early and late hibernation in females (Fig. 3b and Supplementary Table S1).

We next used EM to examine whether the barrier formed by tanycytic end-feet at the ME is altered across hibernation, consistent with a role in regulating GnRH release^19,26^. In females, no significant changes were observed across hibernation, although there was a non-significant trend towards a post-hibernation decrease in the area adjacent to the ME that was occupied by tanycytic end-feet (Supplementary Fig. S2 and Supplementary Table S1). This was concomitant with a post-hibernation increase in neuronal contacts with the ME (Supplementary Fig. S2 and Supplementary Table S1). In contrast, the area occupied by tanycytic end-feet along the ME tended (*p* = 0.09) to increase in males across hibernation (Supplementary Fig. S2 and Supplementary Table S2), and the number of contacts between neurons and the ME decreased significantly (Supplementary Fig. S2 and Supplementary Table S2).

### Activation of the reproductive axis during hibernation

**Fig. 4:**
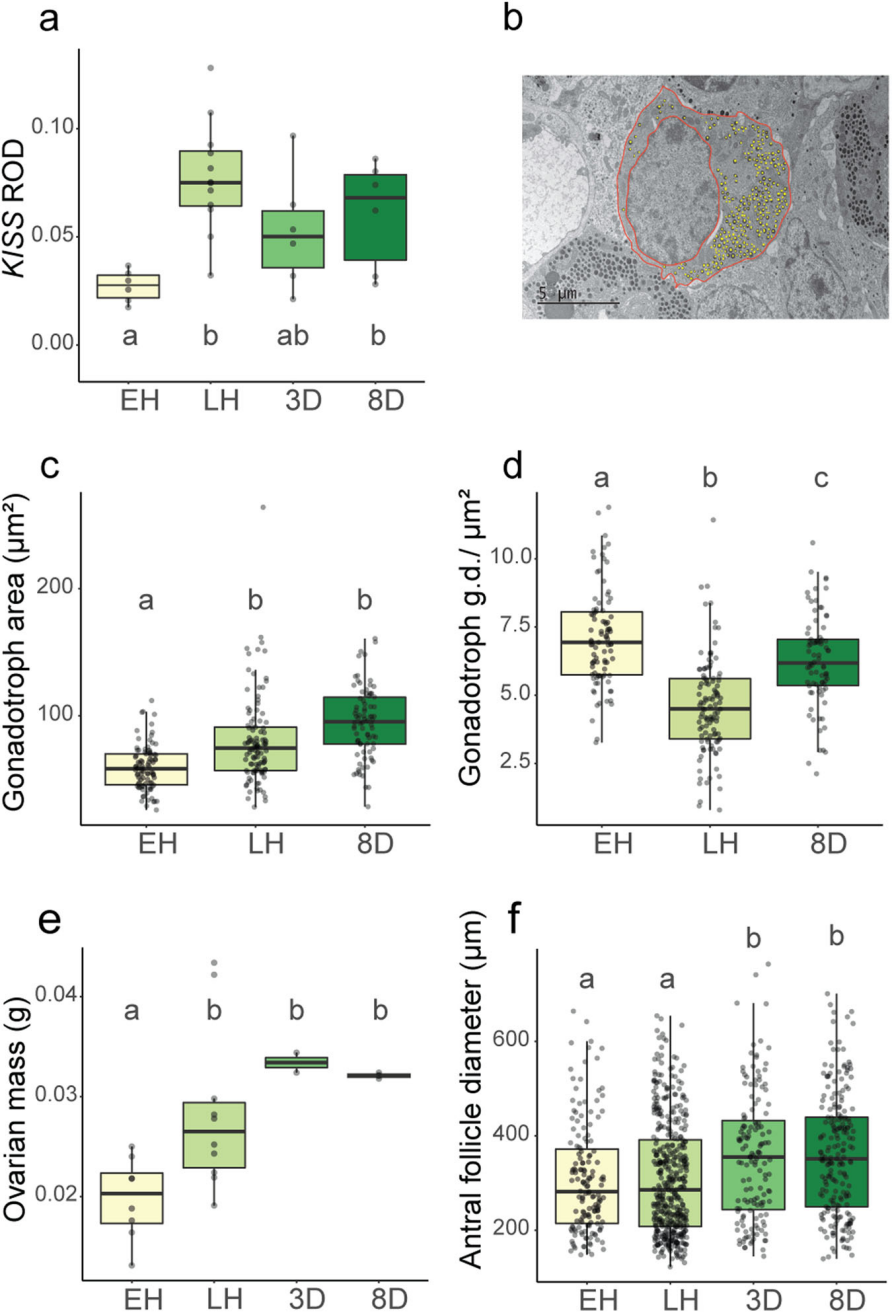
Activation of reproductive axis during hibernation in female arctic ground squirrels. **a** Kisspeptin expression in the arcuate nucleus as quantified by in-situ hybridization (ROD = relative optical density). **b** Electron microscopy image of gonadotrophs with secretory granules in false yellow coloring and cell and nucleus outlined in false pink coloring. **c** Cell area and **d** secretory granule density (g.d.) of gonadotrophs (granules/μm^2^) in the anterior pituitary. **e** Mass of frozen ovaries and **f** diameter of antral follicles. In all graphs, colored shading of box-plots represents distinct sampling groups, lower-case letters indicate significantly different comparisons from data analyzed with a linear mixed effects model (*p* < 0.05), the center line in the box plot represents the median, box boundaries represent the 25th and 75th percentiles, and error bars of boxplots represent the 25th and 75th percentiles ± 1.5 times the interquartile range. For sample sizes, see “Methods”. See also Supplementary Fig. S5.

**Fig. 5:**
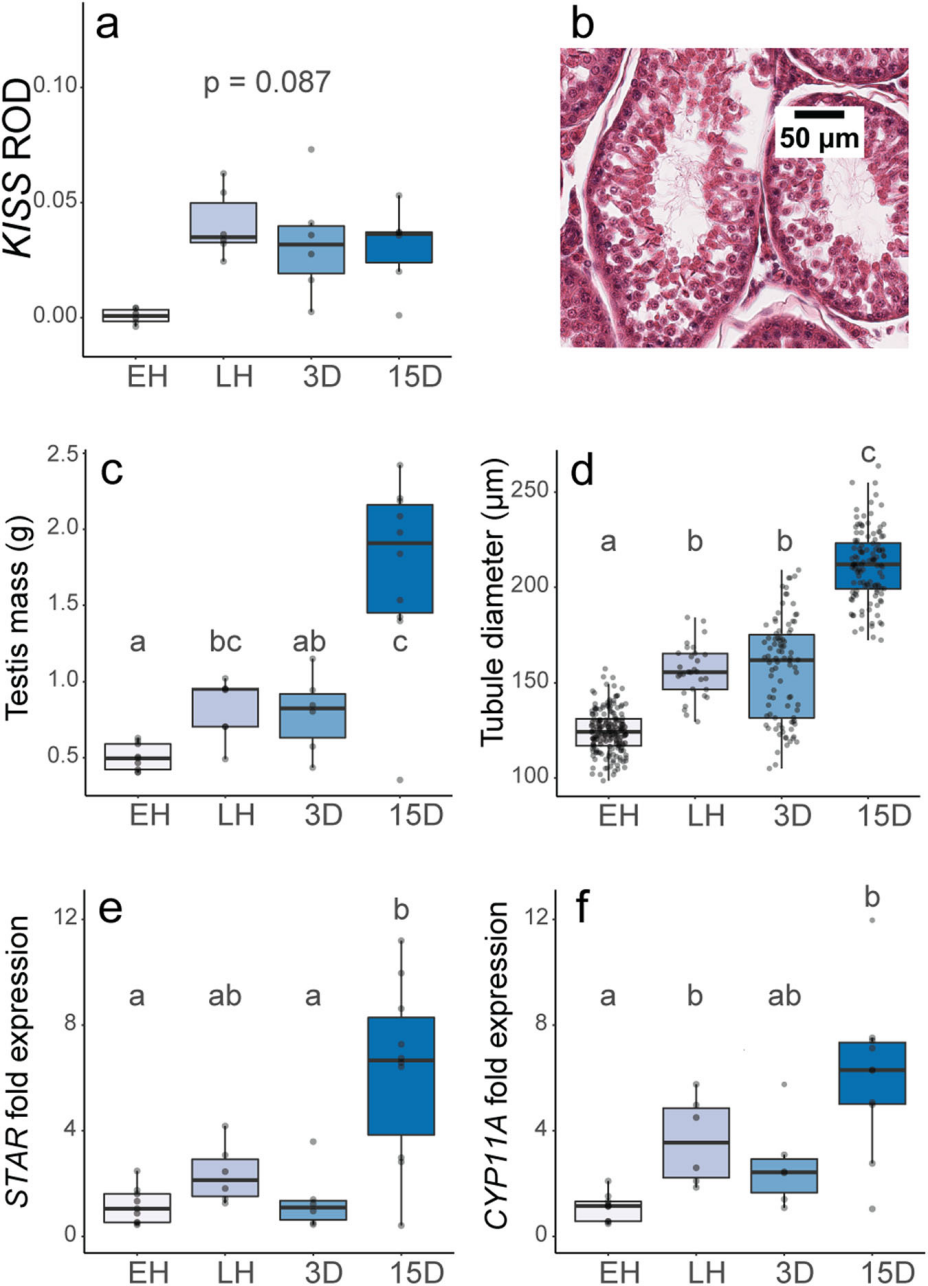
Activation of reproductive axis during hibernation in male arctic ground squirrels. **a** Kisspeptin expression in the arcuate nucleus as quantified by in-situ hybridization (ROD = relative optical density). **b** Seminiferous tubule of 15D post-hibernation male arctic ground squirrel illustrating extrusion of cytosol from spermatids. **c** Mass of frozen testes and **d** diameter of seminiferous tubules in preserved testicular tissue from male arctic ground squirrels. Changes in the expression of steroidogenic genes **e** *STAR* and **f** *CYP11A* relative to early hibernation time points as quantified by qPCR of testicular tissue from male arctic ground squirrels. In all graphs, colored shading of boxplots represents distinct sampling groups, lower-case letters indicate significantly different comparisons from data analyzed with a linear mixed effects model (*p* < 0.05), The center line in the box plot represents the median, box boundaries represent the 25th and 75th percentiles, and error bars of boxplots represent the 25th and 75th percentiles ± 1.5 times the interquartile range. For sample sizes, see “Methods”. See also Supplementary Figs. S1 and S5.

Activation of PT/tanycyte (TSH/DIO) signaling in seasonally breeding mammals is associated with changes in the KISS RFamide system, which is a known TH target and a key regulator of hypothalamic GnRH neurons^39^. The pulsatile release of GnRH into the portal vessels drives reproductive activation by triggering gonadotrophs to synthesize and secrete luteinizing hormone (LH) and follicle-stimulating hormone (FSH). We measured the expression of *Kiss1* in the ARC. This revealed marked suppression in early hibernation, consistent with repression of reproductive function, while in late and post-hibernation we observed significant activation of *Kiss1* expression (Figs. 4a, 5a and Supplementary Tables S1, S2). Accordingly, the changes in the TSH/DIO pathway we observed are also reflected in re-activation of a key hypothalamic neuronal cell type involved in reproduction.

We next examined whether downstream components of the reproductive axis were activated during hibernation. Using EM, we found that morphology of anterior pituitary gonadotrophs changed across hibernation. In females, gonadotroph cell area increased significantly between early and late hibernation (Fig. 4c; Supplementary Tables S1 and S2) while secretory granule density decreased (Fig. 4d; Supplementary Tables S1 and S2). Males showed a similar trend towards decreased secretory granule density across hibernation (Supplementary Fig. S1 and Supplementary Table S2), suggesting that the release of pituitary gonadotropins increases across hibernation. Consistent with these morphological changes in gonadotrophs, ovarian mass in females increased significantly during hibernation and there was a trend towards an increase in the number of antral follicles across hibernation (Fig. 4e; Supplementary Tables S2 and S5). The size of antral follicles, however, did not increase until post- hibernation, when ovarian mass was highest (Fig. 4f; Supplementary Table S1). Nevertheless, quantitative PCR (qPCR) revealed that genes implicated in gonadal growth and maturation, *CYP11A* and *NGF*, showed small (<2 fold), but significant changes in expression in the ovaries between early and late hibernation (Supplementary Fig. S5 and Supplementary Table S1). We did not observe differences in circulating estradiol and progesterone levels across hibernation, although progesterone was significantly lower 3-days post-hibernation relative to all other groups (Supplementary Table S1).

Although changes in the PT/tanycyte (TSH/DIO) axis, PT thyrotrophs, and gonadotrophs of males were muted relative to females, males sampled late in hibernation had larger testes compared to early hibernation males (Fig. 5c, d and Supplementary Table S2). Further, testes exhibited a multi-fold increase in expression of steroidogenic genes *STAR* and *CYP11A* between early and late hibernation, with further increases in expression by 15 days post-hibernation (Fig. 5d, f; Supplementary Table S2). Although testicular growth and steroidogenesis were initiated during hibernation, gamete maturation occurred after hibernation was complete as spermatids and spermatozoa were not visible until 15 days post-hibernation (Supplementary Table S2), when testosterone also increased (Supplementary Table S2). Nevertheless, testicular maturation was incomplete after 2 weeks of post-hibernation euthermia as spermiation was not yet observed.

### Mid-hibernation warming alters TH signaling

**Fig. 6:**
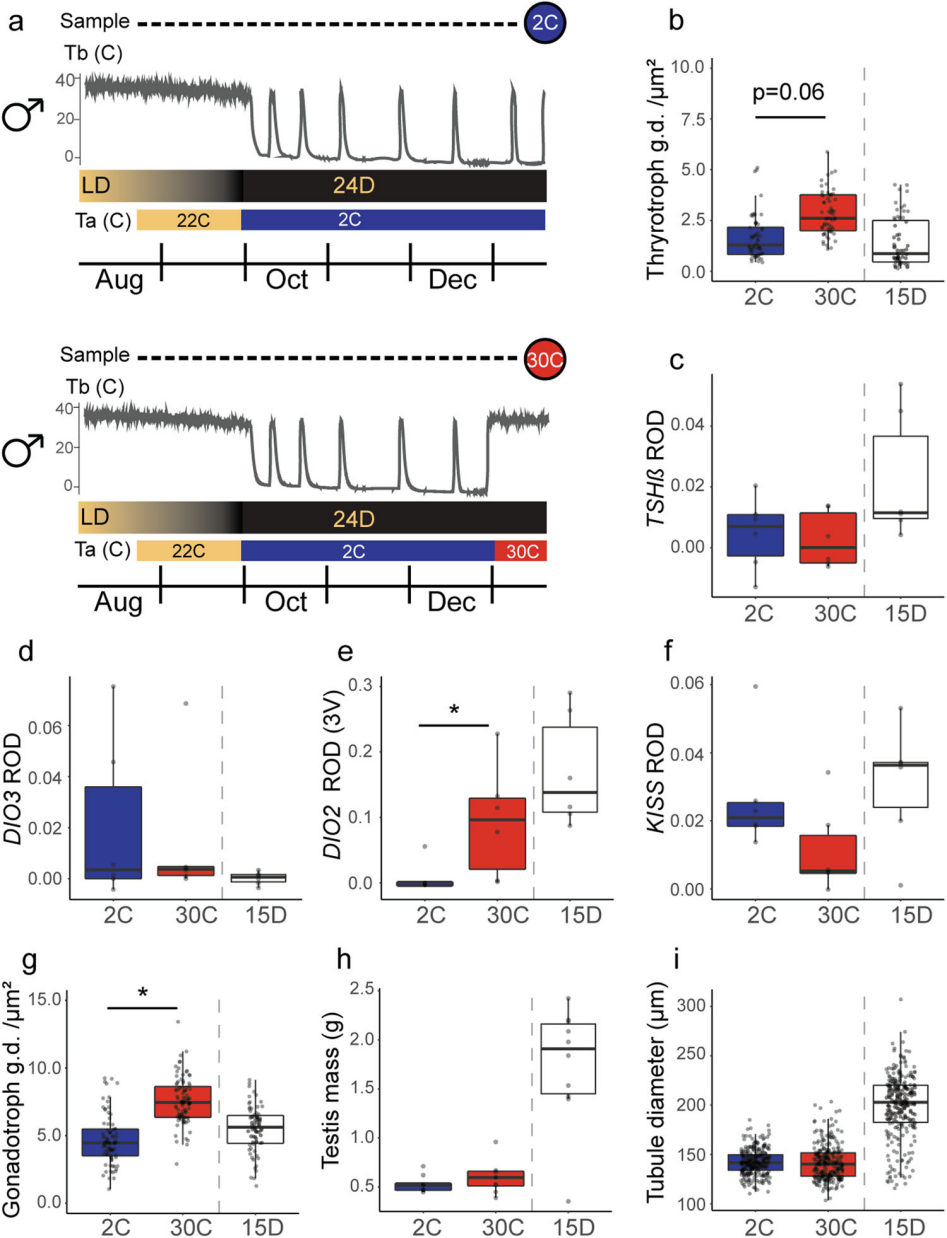
Mid-hibernation warming alters TH signaling. **a** Schematic of experiment testing effects of two-week mid-hibernation warming on activation of TH signaling pathways and the reproductive axis. Filled circles represent the sampling timepoints for control animals sampled at an environmental temperature of 2 °C (top) and experimentally warmed animals sampled at a temperature of 30 °C (bottom). Thin gray lines present expected changes in body temperature across hibernation under temperature treatments; temperature traces are a schematic. The top-colored bar indicates the photoperiodic regime experienced by study subjects (LD = long day, 24D = continuous darkness). The lower colored bar indicates the hibernaculum temperature. Panels **b**–**i** present results for control (blue) and experimentally warmed (red) animals. The center line in the box plot represents the median, box boundaries represent the 25th and 75th percentiles, and error bars represent the 25th and 75th percentiles ± 1.5 times the interquartile range. An additional boxplot (white) shows results for animals 15 days post-hibernation (15D; from the preceding repeated cross-sectional study). Stars indicate significant differences between control and experimental animals (*p* < 0.05) using a linear mixed effects model. For sample sizes, see “Methods”. See also Supplementary Fig. S5.

In our repeated cross-sectional study, we found that the TSH/DIO axis becomes activated during hibernation and, consistent with the known role of TH in stimulating the reproductive axis, we observed concurrent gonadal growth and development. Given that gene transcription and mRNA translation are thought to be globally suppressed during deep torpor in hibernating mammals^40–44^, many of these changes likely occur during periodic arousals. Therefore, we examined the effects of body temperature/metabolism on TSH/DIO circuitry by forcing early resumption of euthermy. This was accomplished by moving a group of males into a warm room (30 °C) mid-hibernation under constant darkness (Fig. 6a). We predicted that warming would alter the ependymal Dio2/Dio3 axis without changing the phase of the circannual cycle in the PT (i.e., PT *TSHß*). The forced termination of hibernation altered some, but not all, aspects of TH signaling. While the expression of *TSHß* (Fig. 6c), *DIO3* (Fig. 6d), and *KISS* (Fig. 6f) were unaffected by treatment, animals that were forced to be euthermic had elevated *DIO2* expression in the ependymal layer of the third ventricle (Fig. 6e; Supplementary Table S3). Secretory granule density in PT thyrotrophs (Fig. 6b and Supplementary Table S3) and anterior pituitary gonadotrophs (Fig. 6f and Supplementary Table S3) also increased, but low levels of *KISS* expression and an absence of testicular growth and development (Fig. 6h, i) indicate that torpor inhibition was insufficient to activate the HPG axis at this stage of hibernation. This is consistent with a hypothalamic thermal sensing mechanism that acts independently of a temperature-compensated circannual clock in the PT^45^, although more work would be required to evaluate this possibility and the other pathways that could regulate tanycytic DIO2 expression.

## Discussion

Our study demonstrates endogenous activation of the retrograde TSH/Deiodinase/T3 signaling pathway during mammalian hibernation in the absence of photic or other environmental input. These changes occurred without detected changes in EYA3, the transcriptional co-activator of TSHß associated with photoperiodic relay and seasonal TH signaling^17,18^. Coupled with this, we observed evidence for cellular remodeling of hypothalamic tanycytes and activation of the reproductive axis. Warming ground squirrels caused premature termination of hibernation and activated thyroid hormone signaling, although this alone was not sufficient to induce reproductive activation. Combined, these data demonstrate the hibernating brain is physiologically dynamic and undergoes preparatory changes in hypothalamic systems regulating reproduction, energy intake, and metabolism. These changes may be directed by an endogenous circannual clock located in the PT^46^, and presumably occurred during arousal bouts when metabolic suppression is periodically lifted.

The centrality of retrograde TH signaling for the control of seasonal cycles has been demonstrated in diverse vertebrate lineages^11,17^. In mammals, changing photoperiod triggers the expression of Eya3, which, in turn activates TSHβ expression in PT thyrotrophs^18^. TSH acts as a seasonal output of the PT, regulating regions of the hypothalamus that control reproductive maturation and metabolism. However, PT thyrotrophs are candidates for an endogenous circannual timing mechanism, operating in a photoperiod- and melatonin-independent manner^46^. Consistent with this concept, blockade of the photoperiodic retinal-pineal relay by pinealectomy does not prevent spontaneous TH signaling or transitions between summer and winter phenotypes in European hamsters (*Cricetus cricetus*) maintained on constant photoperiods^47^. This photorefractory response highlights the importance of melatonin-independent components of endogenous circannual timing systems. Our study extends these findings and reveals that activation of TH signaling occurs across hibernation in the absence of photic input and without changes in EYA3—this activation is likely critically important for hibernating species that transition between seasonal life-history states while sequestered in hibernacula without exposure to photic cues. It has been known for more than 50 years that ground squirrels maintain robust circannual rhythms of hibernation, food consumption, and adiposity while housed in constant darkness, constant light, or constant photoperiod conditions^48,49^. The period length of these free-running circannual rhythms in captive animals is only ~300 days, suggesting circannual and photoperiod-driven melatonin signals converge on PT TSHβ expression during the active season to entrain an individual’s seasonal biological rhythms to the natural environment.

Associated with changes in retrograde TH signaling, we also observed preparatory activation of the reproductive axis while animals were still hibernating. Initiation of seasonal reproductive development and consequent increases in plasma testosterone are a likely mechanism triggering earlier termination of hibernation in males^13^. In support of this hypothesis, castration of male ground squirrels after they have completed hibernation results in torpor re-entry and extension of hibernation^50^, whereas artificial elevation of blood testosterone levels inhibits hibernation even in castrates^51^. Although the mechanisms governing hibernation termination have been understudied in females, we found changes in retrograde TH signaling were amplified in this sex. Field data indicate that reproductive females end hibernation earlier than non-reproductive animals^31^, which is consistent with the limited data indicating estradiol and progesterone may alter the expression of torpor in hibernators^52^. Thus, T3-induced activation of the reproductive axis is likely critical for driving sex-differences in spring hibernation phenology.

Associated with activation of the retrograde TSH/DIO signaling pathway, we also observed pronounced changes across hibernation in Vimentin immuno-labeling and in Nestin expression. Our findings are remarkably consistent with an earlier EM study that revealed profound alterations in the shape and internal cytology of tanycytic processes across hibernation in bats^53^. This suggests that structural remodeling of tanycytes may be a more general feature of hibernation—cytoskeletal plasticity of tanycytes is likely linked to critical roles in regulating seasonal physiology. Activation of TH signaling involves changes in β1-tanycytes, whose processes appear to establish a functional relationship with the PT through the subarachnoid/perivascular space or by direct contacts with PT endocrine cells^54^. Tanycytes also regulate local homeostasis by controlling exchange of signaling molecules, such as glucose and leptin, between the blood and the hypothalamic extracellular fluid^27,28,33^. Access to signaling molecules in the ARC, for example, is dependent on structural changes in tanycytes and junctional complexes at the blood-hypothalamus barrier^34^. Thus, remodeling of tanycytic processes in the ARC may be important for regulating the profound changes in metabolism and appetitive behavior that accompanies the transition from hibernation to springtime activities.

We predicted that we would observe cytoskeletal plasticity of tanycytes, with retraction of tanycytic end feet that ensheath GnRH nerve terminals at the ME allowing for GnRH release as shown in birds and other mammals^19,26^. Unexpectedly, our EM data for males revealed a surprising decrease post-hibernation in contacts between neurons and the ME. This is consistent with a prior study in male Djungarian hamsters (*Phodopus sungorus*) in which exposure to continuous lighting triggers the development of end-feet of tanycytic processes that tightly seal the vascular circumference of the primary plexus of the portal circulation system^55^. Since we were unable to conclusively identify these as GnRH neurons in our study, it may be that seasonal remodeling of tanycytes suppresses neurohormone secretion from another type of neuron, such as TRH and CRH neurons at the ME^56,57^.

An intriguing possibility is that altered Vimentin immuno-staining and Nestin expression across hibernation are associated with seasonal gliogenesis and/or neurogenesis. Although adult neurogenesis occurs predominantly in hippocampal sub-granular and lateral ventricle subventricular zones, the hypothalamus also possesses a neurogenic niche in which tanycytes behave as neural stem cells^58–60^. In seasonal animals, the rate of hypothalamic cell differentiation varies with day length^61,62^, and because these cells can differentiate into many neuronal phenotypes, this has been proposed as a mechanism for seasonal regulation of energy balance and reproduction^63^.

Our findings suggest that seasonal neuroendocrine and cellular remodeling may be one important function of interbout arousals. Arousals are energetically costly—arctic ground squirrels spend only 6% of the hibernation season in arousals, yet this accounts for 86% of energy expenditure when hibernating at 2 °C^64^. The phenomenon of periodic arousal during hibernation is ubiquitous and suggests a critical function despite their high metabolic cost^4,5^. Hypotheses include periodic allowance of gene expression^4^, clearance of metabolic waste products^65^, reduction of sleep debt^66,67^ (but see refs. ^68,69^), regrowth and arborization of hippocampal dendrites that erode across torpor^9^ (but see refs. ^8,70^), and/or activation of the immune system^71^. Since gene transcription and translation are globally suppressed at low body temperatures during torpor, we assume hypothalamic TH signaling, cellular remodeling, and re-programming of brain circuits over the hibernation season accumulated gradually during repeated natural transient re-warming events. Alternatively, the selective transcription and translation of genes critical to seasonal timing may be occurring at low levels during steady state torpor^72,73^, allowing for gradual remodeling of the hibernating brain.

We reveal dynamic changes in the key seasonal neuroendocrine and neural circuitry of the PT and hypothalamus during hibernation, which are compatible with the need for anticipation of the short Arctic spring and summer. The fundamental mechanisms timing these processes, in constant darkness and at low temperatures over the long arctic winter, involve an underlying circannual clock which operates in the absence of external environmental cues^49^.

### Ethics statement

Work with animals was approved by the University of Alaska IACUC under protocol # 864841 and permitted by the Alaska Department of Fish and Game under permits 16-074 and 17-100.

## Materials and methods

Free-living adult and juvenile arctic ground squirrels of both sexes were captured along the Dalton Highway in northern Alaska (68°27’ N, 149°21’ W, elevation 812 m) during July. Squirrels were kept on a 16L: 8D photoperiod at 20 °C and housed individually in 48 × 32 × 32 cm hanging metal cages at University of Alaska Fairbanks. Squirrels were fed 10 pellets of Mazuri rodent chow daily (Land O’Lakes, Inc; St. Louis, MO), had access to ad lib water, and were provided with cotton bedding for nest construction. Beginning August 1st, photoperiod was decreased 30 min a day from 16L: 8D to 4L:20D to mimic declining autumnal photoperiods at high latitudes. When animals exhibited behavioral changes indicating hibernation readiness, they were moved to 2 °C environmental chambers under constant darkness. Once torpid, squirrels were transferred to a plastic tub and provided with gel packs for hydration (HydroGel, ClearH2O, Portland, ME, USA). Squirrels were monitored using the sawdust method^74^ for arousals under dim (<2 lumen) red light.

### Repeated cross-sectional hibernation study: Seasonal changes along the reproductive axis under constant dark and 2 °C

Tissues were sampled from euthermic animals at four timepoints across hibernation: early hibernation, late hibernation, 3d post-hibernation, and 8d post-hibernation (females) or 15d post-hibernation (males). Late hibernation sampling occurred after animals had begun shortening torpor bout length, which occurs prior to terminal arousal. The final sampling point differed by sex (Supplementary Table S4) to reflect naturally occurring sex-differences in behavior post-hibernation. Animals in all groups, except for 8d post-hibernation females, were maintained in continuous darkness from fall hibernation entry until sampling. These females were maintained in continuous darkness until 3d post-hibernation and then transferred to a 16L:8D photoperiod for 5d before being sampled on the eighth day of euthermia.

### Warming experiment: Response of the male reproductive axis to mid hibernation warming

Juvenile males were acclimated to captivity and entered hibernation in an environmental chamber at 2 °C under constant dark conditions as described above for the previous study. On December 26, animals were split randomly into two groups: the control group was maintained under constant darkness at 2 °C and the experimentally warmed group was held at 30 °C also in constant darkness. Experimental animals ended torpor immediately and remained euthermic until sampling. Animals were sampled after 12–16 days.

### Tissue collection

We anesthetized animals deeply using isoflurane, drew up to 5 ml of blood using cardiac puncture, and then euthanized animals using rapid decapitation. Blood was centrifuged and plasma was stored at −80 °C. Brain samples collected for in-situ hybridization were snap frozen on dry ice and stored at −80 °C. Brains collected for immunohistochemistry were placed in Bouin’s fixative (#HT10132, Sigma-Aldrich, St. Louis MO, USA) for 8–12 h, transferred to 70% ethanol, and stored at 4 °C. Brains collected for EM were fixed in 3% paraformaldehyde/0.05% glutaraldehyde in 0.1 M phosphate buffered saline (PBS; pH 7.2) for 24 h. After 24 h, EM samples were transferred to 1:10 dilution of paraformaldehyde/glutaraldehyde fixative in 0.1 M PBS and stored at 4 °C. Gonads from the right side of the body were flash frozen in isopentane on dry ice and stored at −80 °C for quantitative reverse-transcription PCR (qPCR). Gonads from the left side of the body intended for histology were placed in Bouin’s solution for 24 h, washed in 50% and 70% ethanol, and stored in 70% ethanol at 4 °C.

### Immunohistochemistry (IHC)

Preserved whole brains were embedded with wax using a tissue processor (ASP300, Leica Biosystems, Wetzlar, Germany) and mounted in a cassette. Coronal brain sections were sliced at 5 µm on a microtome (RM225, Leica Biosystems), floated on Leica Xtra Adhesive slides (#3800050, Leica Biosystems), dried at 40 *°*C, and stored at 4 *°*C. Immunohistochemistry for vimentin and monocarboxylate transporter 8 (MCT8) with 3,3′-diaminobenzidine (DAB) counterstaining was conducted using a Leica Bond Machine (LBond Rx, Leica Biosystems) and a Bond Polymer Refine Detection Kit (#DS9800, Leica Biosystems). Antibody dilutions were as follows: vimentin 1:500 (#ab8978, Abcam, Cambridge, UK) and MCT8 1:100 (Professor Theo Visser, Erasmus University Medical Center, The Netherlands). Slides were imaged using a 3D Histech P250 Slidescanner (Histech, Budapest, Hungary). Staining of tanycytic projections by vimentin and MCT8 was quantified in the ME and ARC by personnel blind to experimental condition. There were three individuals for each group and two sections per individual were photographed at 3.6× magnification using SigmaScanPro Ver5 (Systat Software, Palo Alto, CA), and a standardized region representing the ARC or ME was defined. A color threshold was used determine the total area occupied by stained pixels and both sections were included in analyses, unless high levels of background staining made thresholding inaccurate or sections were in poor condition.

### In situ hybridization (ISH)

In-situ hybridization was conducted using custom radiolabeled probes for *EYA3*, *TSH-ß*, *DIO2*, *DIO3*, *KISS*, and *NES* mRNA. The *EYA3* (XM_026391640, 4564-5101), *TSHβ* (XM_026394173, 204-422), *DIO2* (XM_026401913, 577-834), *DIO3* (XM_026381476, 96-639) and *KISS* (XM_026397121.1, 1-435) plasmids were cloned as previously described in ref. ^18^ using arctic ground squirrel testes, liver, and/or brown adipose tissue. The *NES* plasmid (XM_026404998, 2231-2650) was cloned using gene synthesis services from GeneArt (Thermo Fisher, CA, USA). Radiolabelled cRNA riboprobes were prepared by plasmid linearization and transcribed using P33 α-UTP (Hartmann-Analytic, Braunschweig, Germany). Fixed sections were hybridized overnight at 60 °C with 15 × 10^5^ cpm of probe per slide. Hybridization signals were visualized on autoradiographic film (Kodak Biomax MR Films, Kodak, USA) after one to two weeks of exposure at −80 °C.

Images taken from exposed film were scored in ImageJ (version 1.52a, National Institutes of Health) and mean pixel grayness was calculated for each region of interest. Staining in the PT was quantified for *EYA3* and *TSH-ß*, staining along the lateral walls of the 3 V as quantified for *DIO3*, and staining in ARC was measured for *KISS*. *DIO2* staining was measured in two separate regions: the first region comprised the lateral walls and base of the 3 V and the second comprised a region along the ME adjacent to the PT. Control regions in the hypothalamus and exposed film were quantified and used to calculate the relative grayness of target regions compared to background staining. There were three individuals for each group except for the late hibernation group of females, which had six individuals. Three sections per individual were included in statistical analyses for targets expressed in the PT, while two slices per individual were included for targets expressed in the hypothalamus.

### Electron microscopy

Tissues were prepared for EM as described in ref. ^75^. Briefly, multiple tissue blocks containing the pituitary, PT, and ME were excised from brains, stained with 1% osmium tetroxide and 2% uranyl acetate, and dehydrated with increasing concentrations of ethanol (70–100%) and acetone. Dehydrated blocks were embedded with Spurr resin (TAAB, Aldermarston, UK). Ultrathin sections (50–80 nm) were taken from resin embedded tissues on a Reichart-Jung Ultracut microtome and mounted on nickel grids (Agar Scientific Ltd; Stanstead, UK). Samples were imaged at 2000× using a JEOL 1010 transmission electron microscope (JOEL USA Inc.; Peabody, MA) using Gatan Microscopy Suite Software (Gatan, Inc., Pleasanton, CA). A subsample of tissues was stained with 2% uranyl acetate and then labeled with LR Gold acrylic resin (London Resin Company Ltd; Reading, UK) to confirm the identity of tanycytes. Tissues were incubated in 1% chicken egg albumin in PBS (EA; Sigma-Aldrich Co. Ltd; Poole, UK) to block non-specific binding sites before sequential incubation with 1:500 vimentin antibody (#ab24525, Abcam, Cambridge, UK) followed by 15 nM protein A gold (diluted 1:60 in PBS/ EA; British Biocell; Cardiff, UK).

There were three individuals per group and for each individual, twenty images of gonadotrophs, thyrotrophs and tanycytes were taken by a photographer blind to treatment and stage of the sample. ImageJ (version 1.52a, National Institutes of Health) was used to quantify the number of secretory granules within PT thyrotrophs and gonadotrophs in the anterior pituitary as well as the total size of these cells. ImageJ was also used to quantify three metrics describing the structure of hypothalamic tanycytes by a single observer that was blind to sample id. Within a 4 μm × 5 μm box (long edge along basal lamina) the observer quantified the area occupied by tanycytes, the number of contacts made between neuron contacts and the median eminence, and the density of ME neuron secretory granules within the box.

### Quantitative reverse-transcription PCR (QRT-PCR)

Frozen ovaries and testes were weighed on an analytical balance (Sartorius, Göttingen, Germany) prior to analysis. RNA was extracted from ovaries and testes using an RNAeasy mini kit (#74106, Qiagen Inc., Valencia, CA, USA). Up to 30 µg of gonadal tissue was homogenized in buffer RLT for 30 s at 3500 rpm with a Bead Bug microtube tissue homogenizer (#D1030, Benchmark Scientific, Edison, NJ, USA). During purification, samples were treated with RNAse-free DNAse (#79254, Qiagen) to avoid genomic DNA contamination. Extracted RNA concentration was measured with a Qubit 4.0 Fluorometer (Invitrogen, Carlsbad, CA, USA) and a Qubit Broad Range Assay Kit (#Q10211, Invitrogen). RNA quality (RIN ≥ 9.4) was confirmed for all samples using an Agilent 2100 bioanalyzer (Agilent Technologies, Santa Clara, CA, USA). cDNA was synthesized by reverse transcribing 1 µg of RNA in a 40 µl reaction with a High Capacity cDNA reverse transcription kit (#436881, Applied Biosystems, Carlsbad, CA, USA) with the following thermal profile: 25 °C (10 min), 37 °C (120 min), 85 °C (5 min), and a 4 °C terminal hold.

Reference and housekeeping primers were designed using the arctic ground squirrel genome (ASM342692v1) on NCBI Primer Blast (for details, see Supplementary Table S5) and previously published primers for which could verify specificity^76^. Samples were assayed in triplicate using Power SYBR™ Green PCR Master Mix (Applied Biosystems) on an ABI-7900 HT system (Applied Biosystems). Each 15 μL reaction contained 3 μL of cDNA in a 1:15 water dilution and was conducted with the following cycle parameters: 95 °C (10 min), 40 cycles of 95 °C f(15 s), and 60 °C (1 min). A final dissociation curve (95 °C for 15 s, 60 °C for 15 s, and 95 °C for 15 s) was run to verify that a single product was amplified. No-template and no-reverse transcription controls were run to ensure reactions were not contaminated. A six-point curve on a 1:5 dilution series of a cDNA pool was run on each plate to calculate reaction efficiency (Supplementary Table S6). Correlation coefficients of curves were 98% or higher. The Pfaffl method^77^ was used to normalize target gene expression relative to reference genes while correcting for reaction efficiency. Sample sizes for qPCR varied by group for males (EH: *n* = 9, LH: *n* = 6, 3D: *n* = 6, 15D: *n* = 10, 2C: *n* = 9, 30C: *n* = 9) and females (EH: *n* = 9, LH: *n* = 17, 3D: *n* = 6, 8D: *n* = 9). Expression levels are reported relative to early hibernation (cross-sectional study) and the control 2 °C group (mid-winter warming experiment).

### Gonad mass

Frozen ovaries and testes were weighed on an analytical balance (Sartorius, Göttingen, Germany) prior to analysis for qPCR (above). Accurate masses could not be obtained for a number of ovarian tissues due to the difficulty of separating surrounding fat tissue from ovary, while keeping the ovary intact. Sample sizes for gonadal mass varied by group: male testes (EH: *n* = 9, LH: *n* = 6, 3d: *n* = 6, 15D: *n* = 10, 2C: *n* = 9, 30C: *n* = 9) and female ovaries (EH: *n* = 8, LH: *n* = 10, 3D: *n* = 2, 8D: *n* = 2).

### HISTOLOGY

Gonads were step-sectioned on a microtome at 100 µm increments and 5 µm thickness sections were stained with hematoxylin and eosin and imaged at 20X (ovaries) and 40X (testes). Slides were scanned using a Leica Aperio ScanScope CS2 digital slide scanner or a Leica CTR6 LED microscope using the Leica program LASX (Leica Biosystems). Sample sizes varied by group for male testes (EH: *n* = 9, LH: *n* = 6, 3d: *n* = 6, 15D: *n* = 19, 2C: *n* = 9, 30C: *n* = 9) and female ovaries (EH: *n* = 9, LH: *n* = 17, 3D: *n* = 6, 8D: *n* = 9). For each testis, 5 sections were scored by a single blind observer^12^: 1- spermatogonia present; 2- spermatogonia and spermatocytes present; 3- spermatids present; 4- spermatozoa present; 5- spermiation with cellular debris present and; 6- gonadal regression. The diameters of six round seminiferous tubules per section (30 tubules per individual) were measured using ImageJ (version 1.52n, National Institutes of Health). The total number of antral follicles containing oocytes were counted in each ovarian section for up to five sections per individual and the diameter of antral follicles containing visible oocytes was measured using ImageJ.

### Hormone assays

Hormones were extracted from 500 µL plasma using solid phase extraction^78^. Extracted samples were eluted in 90% methanol, dried at 35 °C in a ThermoSavant SpeedVac Concentrator (model SDP121P; Thermo Fisher Scientific, Waltham, MA, USA), and stored at −80 °C until use. Samples were resuspended in 1 mL assay buffer (1:2 dilution), shaken for 1 h on a multi-tube vortexer, stored overnight at 4 °C, and then shaken again for 1 h immediately before use.

Commercially available enzyme immunoassay (EIA) kits (Arbor Assays, Ann Arbor, MI, USA) were used to quantify plasma testosterone (kit #K032) in males and estradiol (kit # KB30) and progesterone (kit #K025) in females. Assays were validated for use in arctic ground squirrels using tests of parallelism and accuracy. All assays included a full standard curve and all standards and samples were run in duplicate. Sample sizes varied by group for males (EH: *n* = 9, LH: *n* = 6, 3d: *n* = 6, 15D: *n* = 10, 2C: *n* = 9, 30C: *n* = 9) and females (EH: *n* = 9, LH: *n* = 17, 3D: *n* = 6, 8D: *n* = 9). Intra-assay C.V. of plasma duplicates were as follows: testosterone (2.31 ± 1.99), estradiol (2.79 ± 2.80), progesterone (1.97 ± 1.74). Inter-assay variation was 12.5% (testosterone); 19.2% (estradiol); and 10.1% (progesterone).

### Statistics and reproducibility

Analyses were conducted in R and data for each sex were analyzed separately. Metrics that had multiple measures nested within individual and could be treated as continuous (in-situ data, IHC data, cell size and secretory granule density from EM, antral follicle diameter) were analyzed using a linear mixed effects model in *lme4* with a random effect for individual id and a fixed effect for timepoint or treatment. A random effect for section number was included when appropriate and computationally possible for in-situ and gonad histology data. Results were inspected visually for normality of residuals and heteroscedasticity. When assumptions were violated, data were normalized with *bestNormalize*. Post hoc tests were conducted using *lmerTest* with a Kenward-Roger approximation for degrees of freedom

Metrics for which there was only one measure per individual and/or data were categorical (hormones, qPCR, scored testis staging, gonad mass) were analyzed with a non-parametric Kruskal–Wallace test. A post hoc Dunne Test was used to test pairwise comparisons between groups.

Two measures, ovarian follicle counts and the number of neuron contacts with the basal lamina (EM) were analyzed using a generalized linear mixed effects model with a Poisson distribution with the package *afex*. The model included a random effect for individual id and a fixed effect for timepoint. P-values were estimated using parametric bootstrapping. Post hoc testing for all pairwise comparisons was conducted using *emmeans*.

### Reporting summary

Further information on research design is available in the Nature Research Reporting Summary linked to this article.

## Supplementary information

Supplementary Information

Reporting Summary

## Data Availability

Raw data and images are available from the Dryad repository: 10.25338/B8QD1H

## Code availability

Custom R scripts for data analysis are available from the authors upon request.
