# Supplementary material for: Hypothalamic remodeling of thyroid hormone signaling during hibernation in the arctic ground squirrel
Source: Commun Biol. 2022 May 23;5:492. doi: 10.1038/s42003-022-03431-8 (PMC9126913; doi:10.1038/s42003-022-03431-8)
Supplement: Supplementary file 1 — Supplementary Information [file 42003_2022_3431_MOESM1_ESM.pdf]

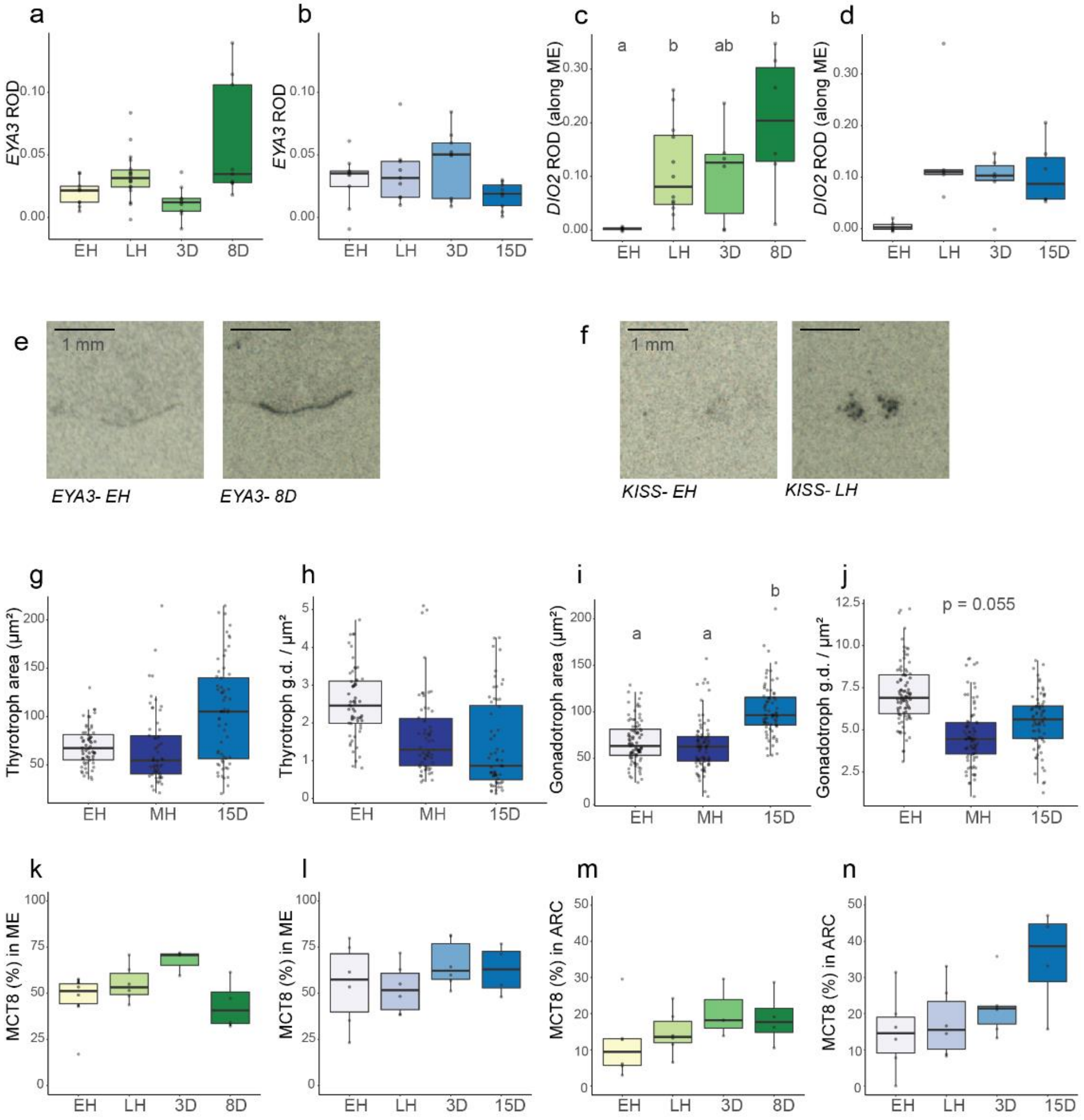

Fig S1. Additional comparisons for cross-sectional hibernation study in arctic ground squirrels. *EYA3* mRNA expression in the pars tuberalis of females (a) and males (b) and *DIO2* mRNA expression along the median eminence in females (c) and males (d) as measured by in situ hybridization. Expression of EYA3 (e) and KISS (f) mRNA in female arctic ground squirrels. Photos selected to represent highest expression observed in group and scale bars are 1mm. Changes in the cross-sectional area and granule density (g.d.) of thyrotrophs (g,h) and gonadotrophs (i,j) in male arctic ground squirrels based on electron microscopy imaging. Panels g-j include a mid-hibernation (MH) control group, which is the control group from the mid-hibernation warming study. Changes in staining of MCT8 protein levels visualized by immunohistochemistry in females (k,m) and males (l,n). For all plots, significantly different pairwise comparisons with linear-mixed effects modelling are indicated by lower-case letters ($p<0.05$). The center line in the box plot represents the median, box boundaries represent the 25th and 75th percentiles, and error bars represent the 25th and 75th percentiles ± 1.5 times the interquartile range and colored shading represents sampling groups. For sample size, see methods. See also Figures 1, 2, 3, and 5.

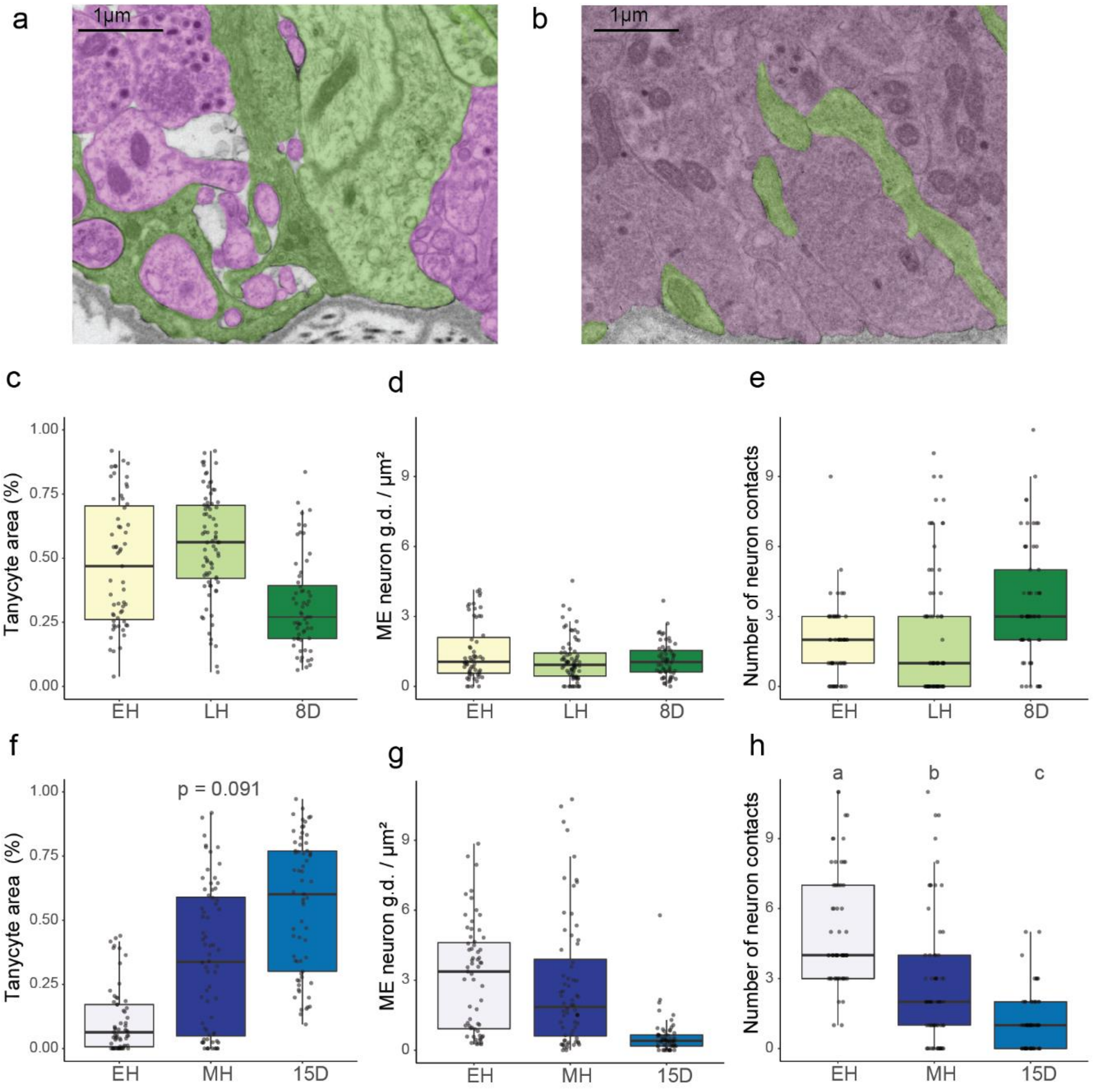

Fig S2. Sex-differences in tanycytic remodeling during hibernation under constant darkness. Sex-differences in tanycytic remodeling during hibernation under constant darkness. a and b) Electron microscopy (EM) images of tanycytes (green false shading) illustrating the access of neurons (purple false shading) to the median eminence (image scale H: 4μm x W: 5μm). In a, tanycytic endfeet enclose neurons preventing access to the median eminence and release of secretory granules. In b, tanycytic endfeet retract, permitting neuron access to the median eminence and granule release. Quantification of the cross-sectional area (μm^2) of tanycytic processes at the median eminence (c) secretory granule density (g.d.) in neurons at the median eminence (d), and number of contacts between neurons and the median eminence (e) in EM images in female arctic ground squirrels. Panels f-h show corresponding data in males. Male data include a mid-hibernation (MH) group, which is the control group from mid-hibernation warming study. These animals and tissues were treated identically in the two studies. Significantly different pairwise comparisons with linear-mixed effects modelling are indicated by letters ($p<0.05$). The center line in the box plot represents the median, box boundaries represent the 25th and 75th percentiles, and error bars represent the 25th and 75th percentiles ± 1.5 times the interquartile range. For sample size, see methods.

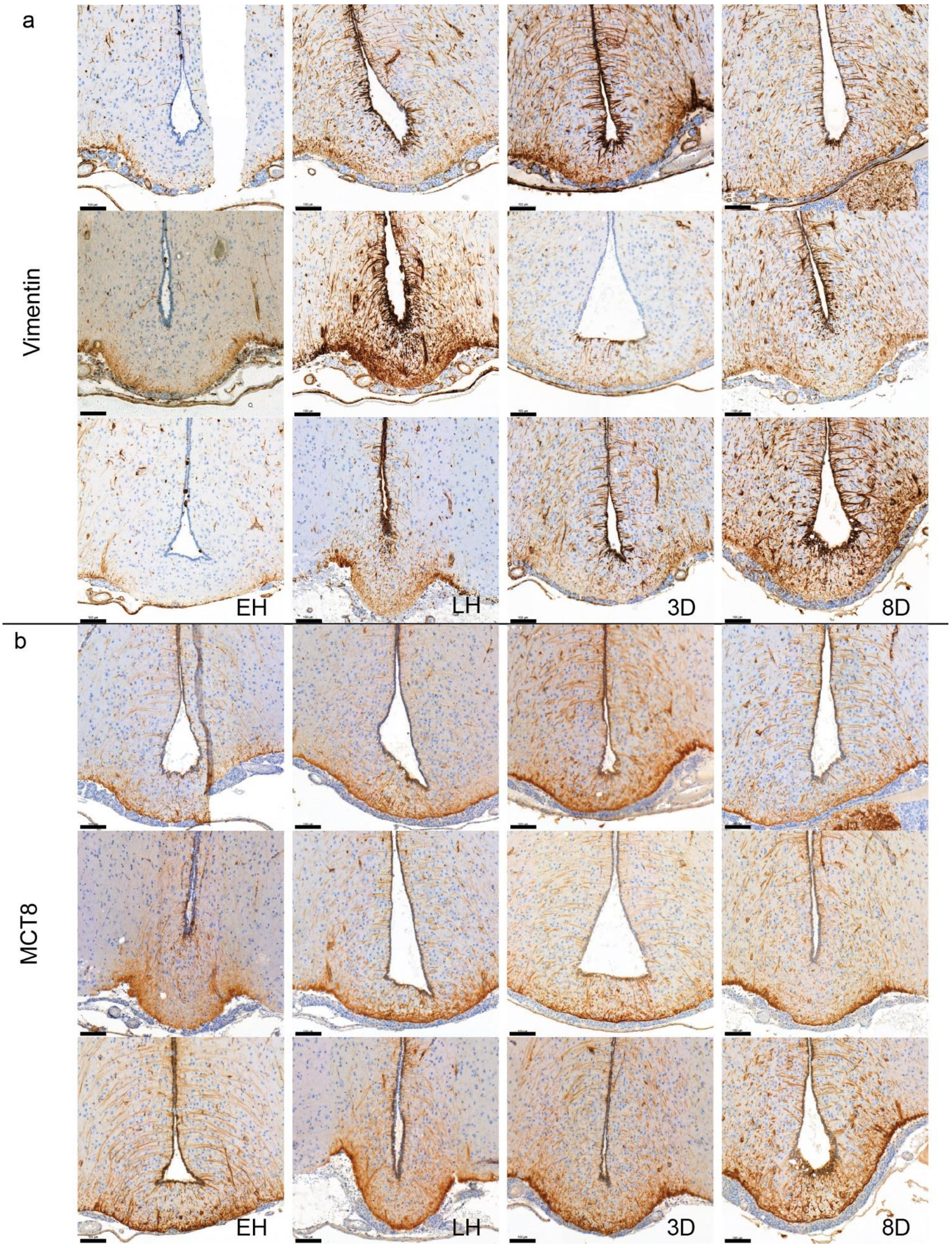

Fig S3. Vimentin and MCT8 immunohistochemistry in female arctic ground squirrels. a) DAB immunohistochemistry for vimentin and b) MCT8 in all female arctic ground squirrels. Columns represent the timepoint of sampling with each image representing a unique individual. Scale bars are 100 µm. See also Figure 3.

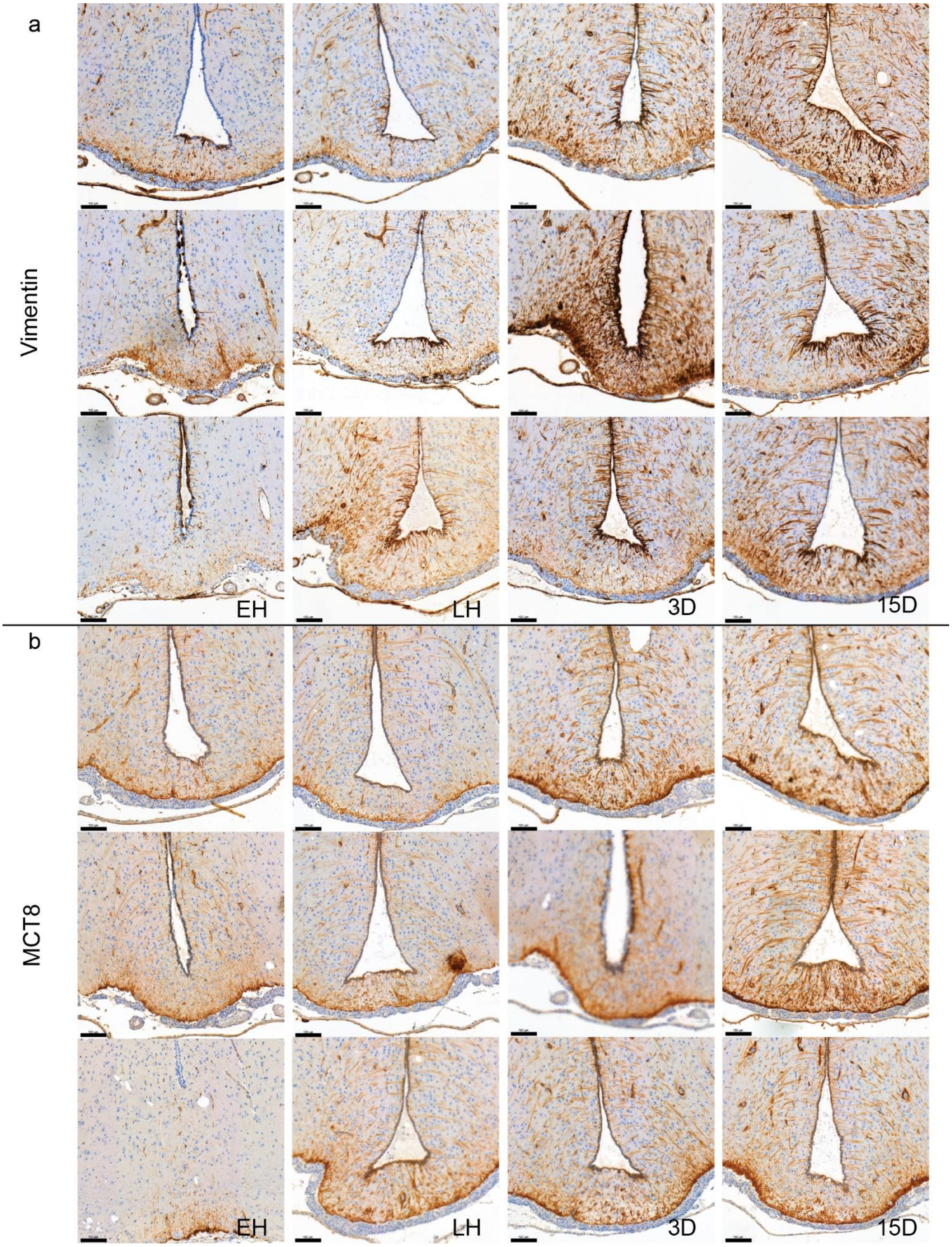

Fig S4. Vimentin and MCT8 immunohistochemistry in male arctic ground squirrels. a) DAB immunohistochemistry for vimentin and b) MCT8 in all male arctic ground squirrels. Columns represent the timepoint of sampling with each image representing a unique individual. Scale bars are 100 µm. See also Figure 3.

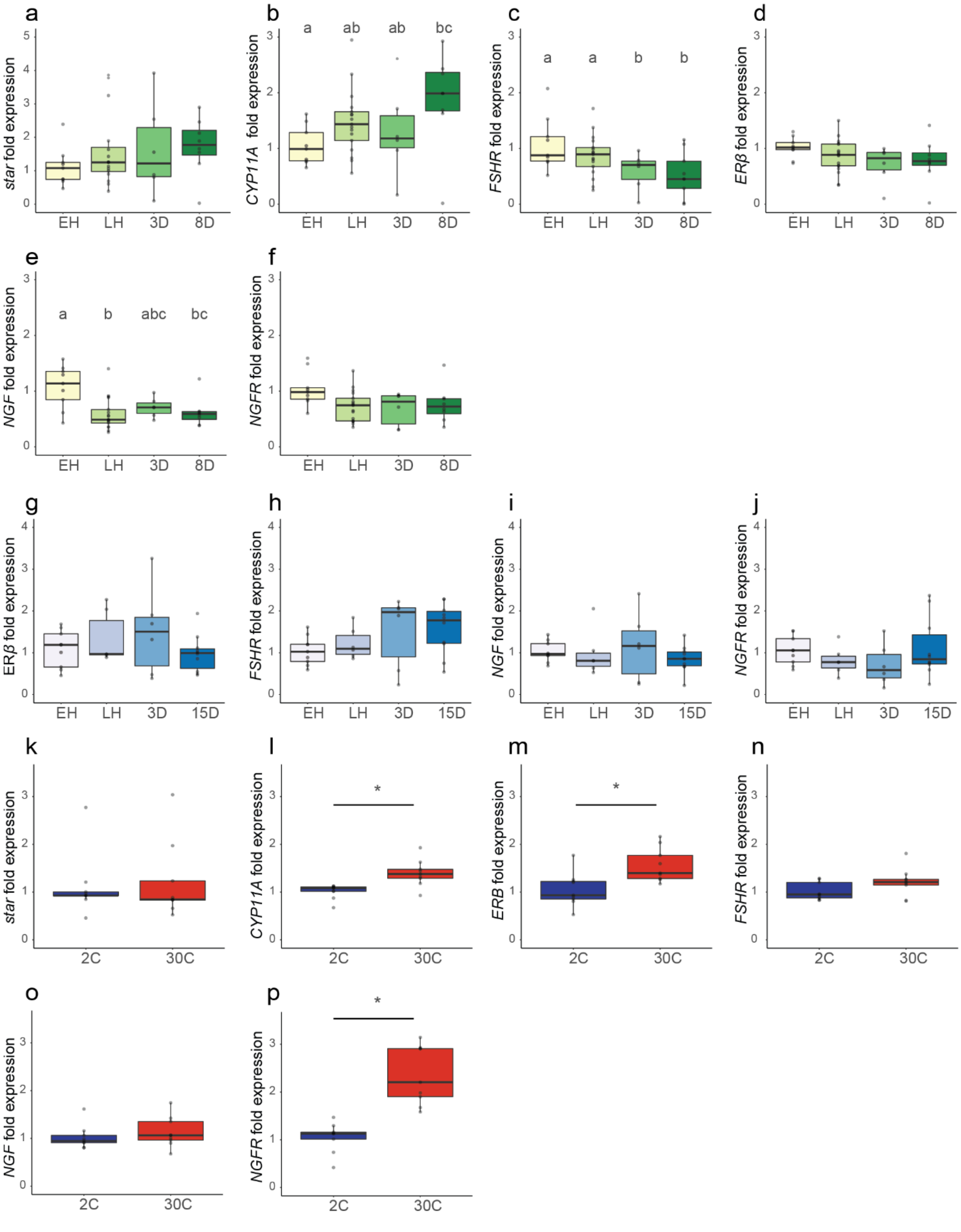

Fig S5. Quantitative PCR of ovaries and testes. Panels a-f show fold changes in steroidogenic gene expression in ovaries collected from female arctic ground squirrels across hibernation relative to the EH (early hibernation) group. Panels g-j show fold changes in steroidogenic gene expression in testes from male arctic ground squirrels across hibernation relative to the EH group. Panels k-p show fold changes in steroidogenic gene expression in testes in control (2°C) and artificially warmed (30°C) groups relative to the control group. Significantly different pairwise comparisons with linear-mixed effects modelling are indicated by lower-case letters ($p<0.05$) for panels a-j and stars for panels k-p. The center line in the box plot represents the median, box boundaries represent the 25th and 75th percentiles, and error bars represent the 25th and 75th percentiles ± 1.5 times the interquartile range. For sample size, see methods. See also Figures 4, 5, and 6.

Table S1: Omnibus test results for all data collected from female arctic ground squirrels during the cross-sectional hibernation study. The symbol (*) indicates that the variance attributed to the random effect (RE) was near zero and could not be estimated.

**Model Type: LMM with Gaussian Distribution**								
**Target**	**RE**	**Transformation**	**Sum.Sq**	**Mean.Sq**	**NumDF**	**DenDF**	**F**	**P(F)**
EYA3	id	None	0.001	0	3	11	1.998	0.173
TSHB	id*	Ordered normal	24.737	8.246	3	11	17.783	0
DIO2- 3V	id	Ordered normal	2.687	0.896	3	11	4.944	0.021
DIO2- along ME	id	Ordered normal	7.618	2.539	3	11	4.977	0.02
DIO3	id *	Ordered normal	14.467	4.822	3	11	8.773	0.003
KISS	id	Ordered normal	11.109	3.703	3	11	6.168	0.01
NES	id	Ordered normal	7.364	2.455	3	11	9.54	0.002
Thyrotroph cell area	id	Ordered normal	4.541	2.27	2	7	3.21	0.103
Thyrotroph granule density	id	Ordered normal	4.354	2.177	2	7	6.05	0.03
Tanycytes- ME percent Area	id	Ordered normal	1.687	0.843	2	6.998	1.673	0.255
ME neuron granule density	id	Ordered normal	0.102	0.051	2	6.999	0.103	0.903
Vimentin (ARC)	id	None	515.722	171.907	3	7.49	5.881	0.022
Vimentin (ME)	id	Ordered normal	4.291	1.43	3	7.761	5.332	0.027
MCT8 (ARC)	id	None	187.403	62.468	3	6.637	0.997	0.451
MCT8 (ME)	id	None	506.292	168.764	3	6.974	1.826	0.231
Gonadotroph cell area	id	Arc sin	15.353	7.677	2	6.959	10.474	0.008
Gonadotroph granule density	id	none	50.213	25.107	2	6.751	35.502	0
Antral follicle diameter	id/section	Ordered normal	259114.1	86371.35	3	35.925	6.05	0.002
**Model Type: LMM with Poisson Distribution**								
**Target**	**RE**	**Transformation**	**Chisq**	**df**	**P(PB)**			
Number of antral follicles	id/section	None	7.120	3	0.073			

Number of ME neuron contacts	id	None	3.151	2	0.328			
**Model Type: Kruskall- Wallis Test**								
**Target**	**RE**	**Transformation**	**Kruskall-Wallis Chi-Sq.**	**df**	**p-value**			
STAR fold expression	None	None	3.363	3	0.339			
CYP11A fold expression	None	None	9.005	3	0.029			
ERß fold expression	None	None	5.108	3	0.164			
FSHR fold expression	None	None	7.946	3	0.047			
NGF fold expression	None	None	10.377	3	0.016			
NGFR fold expression	None	None	6.834	3	0.077			
Ovarian mass	None	None	12.120	3	0.007			
Progesterone	None	None	9.677	3	0.022			
Estradiol	None	None	4.020	3	0.259			

Table S2: Omnibus test results for all data collected from male arctic ground squirrels during the cross-sectional hibernation study. The symbol (*) indicates that the variance attributed to random effect (RE) was near zero and could not be estimated. The symbol (&) denotes electron microscopy analyses for which the control group from the mid-hibernation warming study was also included in analyses.

Target	RE	Transformation	Sum.Sq	Mean.Sq	NumDF	DenDF	F	P(F)
EYA3	id and position	None	0.001	0	3	8	0.99	0.445
TSHB	id and position	None	0.001	0	3	8	2.179	0.168
DIO2- 3V	id and position	None	0.009	0.003	3	8.001	2.717	0.115
DIO2- along ME	id	Square root	3.336	1.112	3	7.967	5.164	0.028
DIO3	id and position	Ordered normal	3.2	1.067	3	8	1.09	0.407
KISS	id and position	Ordered normal	2.383	0.794	3	8	3.133	0.087
NES	id and position	Ordered normal	2.046	0.682	3	8	2.062	0.184
Thyrotroph cell area (&)	id	Ordered normal	0.574	0.287	2	6	0.686	0.539
Thyrotroph granule density (&)	id	Ordered normal	1.509	0.754	2	6	1.641	0.27
Gonadotroph cell area (&)	id	Ordered normal	6.877	3.438	2	5.997	5.876	0.039
Gonadotroph granule density (&)	id	Ordered normal	5.997	2.999	2	5.997	4.894	0.055
Tanycytes- ME percent Area (&)	id	Ordered normal	2.314	1.157	2	6	3.674	0.091
ME neuron granule density (&)	id	Ordered normal	1.514	0.757	2	6	2.069	0.207
Vimentin (ARC)	id	Arcsin	2.23	0.743	3	7.977	4.357	0.043
Vimentin (ME)	id	Ordered normal	0.629	0.21	3	7.991	1.045	0.424
MCT8 (ARC)	id	None	828.335	276.112	3	7.937	2.865	0.104
MCT8 (ARC)	id	Ordered normal	3.43	1.143	3	7.946	1.257	0.353

Tubule diameter	id/section	Ordered normal	11.276	3.759	3	26.002	20.598	0
**Model Type: LMM with Poisson Distribution**								
**Target**	**RE**	**Transformation**	**Chisq**	**df**	**P(PB)**			
Number of ME neuron contacts (&)	id	None	9.835	2	0.035			
**Model Type: Kruskall- Wallis Test**								
**Target**	**RE**	**Transformation**	**Kruskall Wallis Chi-Sq.**	**df**	**p-value**			
STAR fold expression	None	None	13.811	3	0.003			
CYP11A fold expression	None	None	17.008	3	0.001			
ERß fold expression	None	None	1.145	3	0.766			
FSHR fold expression	None	None	4.587	3	0.205			
NGF fold expression	None	None	2.034	3	0.565			
NGFR fold expression	None	None	3.055	3	0.383			
Testis mass	None	None	17.185	3	0.001			
Testis staging	None	None	24.024	3	0.000			
Testosterone	None	None	9.061	3	0.028			

Table S3: Omnibus test results for data collected from male arctic ground squirrels during the mid-hibernation warming experiment. The symbol (*) indicates that the variance attributed to the random effect (RE) was near zero and could not be estimated.

**Target**	**RE**	**Transformation**	**Sum.Sq**	**Mean.Sq**	**NumDF**	**DenDF**	**F**	**P(F)**
EYA3	id*	Ordered normal	0.391	0.391	1	3.899	0.382	0.571
TSHß	id*	Ordered normal	0.006	0.006	1	4	0.006	0.942
DIO2- 3V	id	Ordered normal	4.465	4.465	1	4	10.604	0.031
DIO2- along ME	id	Ordered normal	0.34	0.34	1	4	0.755	0.434
DIO3	id	Ordered normal	0.001	0.001	1	4	0.002	0.965
KISS	id	Ordered normal	0.773	0.773	1	4	2.117	0.219
NES	id	None	0	0	1	4	0.196	0.681
Thyrotroph cell area	id	Ordered normal	0.132	0.132	1	4	0.197	0.68
Thyrotroph granule density	id	Ordered normal	3.84	3.84	1	4	6.394	0.065
Tanycytes- ME percent Area	id	Ordered normal	0.065	0.065	1	4	0.209	0.671
ME neuron granule density	id	Ordered normal	0.518	0.518	1	4	1.368	0.307
Vimentin (ARC)	id	Ordered normal	0.58	0.58	1	4	0.697	0.451
Vimentin (ME)	id	Ordered normal	2.014	2.014	1	4	3.993	0.116
MCT8 (ARC)	id	None	54.926	54.926	1	2.949	3.207	0.173
MCT8 (ARC)	id	None	7.493	7.493	1	2.907	0.14	0.734
Gonadotroph cell area	id	Ordered normal	3.001	3.001	1	3.988	3.558	0.133
Gonadotroph granule density	id	Ordered normal	27.859	27.859	1	3.929	47.002	0.003
Tubule diameter	id/section	Yeo-Johnson	0.036	0.036	1	16	0.071	0.793
Lumen diameter	id/section	Box-Cox	1.065	1.065	1	16	1.66	0.216
Table continued on next page								

Model Type: LMM with Poisson Distribution								
**Target**	**RE**	**Transformation**	**Chisq**	**df**				
Number of ME neuron contacts	id	None	0.066	1	0.851			
**Model Type: Kruskall- Wallis Test**								
**Target**	**RE**	**Transformation**	**Kruskall Wallis Chi-Sq.**	**df**	**p-value**			
STAR fold expression	None	None	0.563	1	0.453			
CYP11A fold expression	None	None	8.751	1	0.003			
ERß fold expression	None	None	7.253	1	0.007			
FSHR fold expression	None	None	0.860	1	0.354			
NGF fold expression	None	None	1.031	1	0.310			
NGFR fold expression	None	None	12.789	1	0.000			
Testis mass	None	None	1.031	1	0.310			
Testis staging	None	None	NA	1	NA			
Testosterone	None	None	0.563	1	0.453			

Table S4: Sampling of arctic ground squirrels for repeated cross-sectional study.

**Timepoint**	**Sampling dates**	**Days after hibernation entry (mean ± SD)**	**Photoperiod during hibernation**
MALES			
Early hibernation	10/6 - 11/20	18 ± 13	24D
Late hibernation	2/27 - 3/15	141 ± 22	24D
3d post-hibernation	2/27 - 4/6	152 ± 31	24D
15d post-hibernation	3/9 - 4/17	138 ± 36	24D
FEMALES			
Early hibernation	8/16- 10/24	7 ± 2	24D
Late hibernation	3/14 - 3/27	171 ± 12	24D
3d post-hibernation	2/3- 4/8	161 ± 23	24D
8d post-hibernation	3/28- 4/21	182 ± 22	24D until 3 days post-hibernation, transferred to 16L:8D for final 5 days before sampling.

Table S5: Primers for qRT-PCR in arctic ground squirrel gonadal tissue. Six reference genes were evaluated for expression stability (i.e. no differences across timepoints and Ct standard deviation below one). When more than three met these criteria for a tissue, the R package ctrlGene and geNorm algorithm were used to identify the three most stably expressed genes. For tissues with two or more stable reference genes, the geometric mean was used as a reference. For the cross-sectional study, *B2M* was used as a reference gene for testes whereas *YWHAZ* and *HPRT* were used for ovaries. For the mid-winter warming experiment, *ACTB*, *PPIA*, and *HPRT* were used.

Gene Symbol	Protein	Direction	Sequence	Product Length
Reference Genes				
B2M	beta-2-microglobulin	Forward	GCAGTCGGACCTCTCTTTCA	146
		Reverse	TGTCTCGGTCCCACTTCACT	
ACTB	Actin, cytoplasmic like 1	Forward	GCCTGCGTTCTGTTCTTTTT	149
		Reverse	TACATGGCCGGTGTGTTGAA	
HPRT	hypoxanthine phosphoribosyltransferase 1	Forward	CTTGCTGGTGAAAAGAACCTCTA	98
		Reverse	AGTCAAGGGCATATCCAACAAC	
PPIA	peptidylprolyl isomerase A	Forward	AGGCCCTGGCATTTTGTCTA	97
		Reverse	CTTGCCATCCAACCACTCAG	
RSP3	ribosomal protein S3	Forward	CGAGTTACACCAACCAGGACA	124
		Reverse	CAGGGAAGCCAAACCTCCTCT	
YWHAZ	protein kinase C inhibitor protein	Forward	GGGCTAAGTTATACCCAAAGC	147
		Reverse	AGAAAACATTGTCCCTGCTC	
Genes of Interest				
CYP11A	cytochrome P450 side chain cleavage	Forward	AGACACTGAGACTCCACCC	183
		Reverse	CATTGTCCAGCCATCGGTTT	
ERß	estrogen receptor 2/ estrogen receptor beta	Forward	ATGGAGTCTGGTCGTGTGAA	90
		Reverse	ACTGATTCGTAGCTGGGCAA	
FSHR	follicle stimulating hormone receptor	Forward	TTTGCAAACTGGAGACGGC	105
		Reverse	GACTCTCTGACCCCTAGCCT	
NGF	nerve growth factor	Forward	GGCCCAATAACAGCTTTTCCA	120
		Reverse	CTGCCTGTATGCCGATCAAAA	
NGF	nerve growth factor receptor	Forward	CCAGACTGTGTGTGAACCCT	178
		Reverse	GGTGATCTCGTCCTGGTAGT	
STAR	steroidogenic acute regulatory protein	Forward	AACCCAGCAGGCAAATGGAG	131
		Reverse	CCTCCATGCGTTCCACAAGT	

Table S6: Reaction efficiencies for qRT-PCR of arctic ground squirrel testicular and ovarian tissue.

**Target**	**Efficiency in Testes**	**Efficiency in Ovaries**
Reference Genes		
B2M	105%	109%
ACTB	107%	Not run
HPRT	105%	108%
PPIA	101%	Not run
RSP3	105%	110%
YWHAZ	106%	112%
Genes of Interest		
CYP11A	107%	107%
ERß	NA	112%
FSHR	111%	116%
NGF	112%	117%
NGFR	103%	104%
STAR	114%	115%
