# Supplementary material for: Hypothalamic remodeling of thyroid hormone signaling during hibernation in the arctic ground squirrel
Source: Commun Biol. 2022 May 23;5:492. doi: 10.1038/s42003-022-03431-8 (PMC9126913; doi:10.1038/s42003-022-03431-8)
Supplement: Supplementary file 2 — Reporting Summary [file 42003_2022_3431_MOESM2_ESM.pdf]

nature portfolio

Corresponding author(s): Chmura and Williams

Last updated by author(s): Apr 12, 2022

Reporting Summary

Nature Portfolio wishes to improve the reproducibility of the work that we publish. This form provides structure for consistency and transparency in reporting. For further information on Nature Portfolio policies, see our Editorial Policies and the Editorial Policy Checklist.

Statistics

For all statistical analyses, confirm that the following items are present in the figure legend, table legend, main text, or Methods section.

n/a	Confirmed	
☐	☒	The exact sample size (n) for each experimental group/condition, given as a discrete number and unit of measurement
☐	☒	A statement on whether measurements were taken from distinct samples or whether the same sample was measured repeatedly
☐	☒	The statistical test(s) used AND whether they are one- or two-sided<br>*Only common tests should be described solely by name; describe more complex techniques in the Methods section.*
☐	☒	A description of all covariates tested
☐	☒	A description of any assumptions or corrections, such as tests of normality and adjustment for multiple comparisons
☒	☐	A full description of the statistical parameters including central tendency (e.g. means) or other basic estimates (e.g. regression coefficient) AND variation (e.g. standard deviation) or associated estimates of uncertainty (e.g. confidence intervals)
☐	☒	For null hypothesis testing, the test statistic (e.g. F, t, r) with confidence intervals, effect sizes, degrees of freedom and P value noted<br>*Give P values as exact values whenever suitable.*
☒	☐	For Bayesian analysis, information on the choice of priors and Markov chain Monte Carlo settings
☐	☒	For hierarchical and complex designs, identification of the appropriate level for tests and full reporting of outcomes
☒	☐	Estimates of effect sizes (e.g. Cohen's d, Pearson's r), indicating how they were calculated

Our web collection on statistics for biologists contains articles on many of the points above.

Software and code

Policy information about availability of computer code

Data collection	Data were collected using Image J (verion 1.52a), SigmaScanPro Ver5, and Gatan Microscopy Suite Software
Data analysis	Data were analyzed in R version 3.6.1.

For manuscripts utilizing custom algorithms or software that are central to the research but not yet described in published literature, software must be made available to editors and reviewers. We strongly encourage code deposition in a community repository (e.g. GitHub). See the Nature Portfolio guidelines for submitting code & software for further information.

Data

Policy information about availability of data

All manuscripts must include a data availability statement. This statement should provide the following information, where applicable:

- Accession codes, unique identifiers, or web links for publicly available datasets
- A description of any restrictions on data availability
- For clinical datasets or third party data, please ensure that the statement adheres to our policy

Data are available from the Dryad repository: https://doi.org/10.25338/B8QD1H

Field-specific reporting

Please select the one below that is the best fit for your research. If you are not sure, read the appropriate sections before making your selection.

☒ Life sciences ☐ Behavioural & social sciences ☐ Ecological, evolutionary & environmental sciences

For a reference copy of the document with all sections, see nature.com/documents/nr-reporting-summary-flat.pdf

Life sciences study design

All studies must disclose on these points even when the disclosure is negative.

Sample size	Sample sizes for tissue collection were determined based on results from previous studies in other taxa.
Data exclusions	Data were only excluded when there was a biological basis for that decision (e.g. section of brain tissue failed to capture the brain region of interest or was damaged).
Replication	This study has been conducted only once and we are facilitating future replication by a thorough reporting of methodological details in this manuscript.
Randomization	Random
Blinding	Scorers for gonad histology and electron microscopy were blind to conditions. For other measures, blinding was not relevant because scores were quantitative and not subject to bias (i.e. automatically calculated by a computer).

Reporting for specific materials, systems and methods

We require information from authors about some types of materials, experimental systems and methods used in many studies. Here, indicate whether each material, system or method listed is relevant to your study. If you are not sure if a list item applies to your research, read the appropriate section before selecting a response.

Materials & experimental systems

n/a	Involved in the study	
☐	☒	Antibodies
☒	☐	Eukaryotic cell lines
☒	☐	Palaeontology and archaeology
☐	☒	Animals and other organisms
☒	☐	Human research participants
☒	☐	Clinical data
☒	☐	Dual use research of concern

Methods

n/a	Involved in the study	
☒	☐	ChIP-seq
☒	☐	Flow cytometry
☒	☐	MRI-based neuroimaging

Antibodies

Antibodies used	Abcam vimentin antibody #ab8978 and #ab24525. MCT8 antibody was donated by Professor Theo Visser, Erasmus University Medical Center, The Netherlands.
Validation	Abcam # ab8978 was validated using knockout validation. Information about abcam #24525 can be found at the following link: https://www.abcam.com/vimentin-antibody-ab24525.html.

Animals and other organisms

Policy information about studies involving animals; ARRIVE guidelines recommended for reporting animal research

Laboratory animals	NA
Wild animals	Free-living adult and juvenile arctic ground squirrels (Urocitellus parryii) were live captured along the Dalton Highway corridor in northern Alaska near the Atigun River (68°27′ N, 149°21′ W, elevation 812 m) during July of 2016 and 2017. Animals were captured using carrot-baited Tomahawk traps. All animals were lethally sampled during the course of the study using rapid decapitation under deep sedation with isoflurane.
Field-collected samples	NA
Ethics oversight	Work with animals was approved by the University of Alaska IACUC under protocol # 864841 and permitted by the Alaska Department of Fish and Game under permits 16-074 and 17-100.

Note that full information on the approval of the study protocol must also be provided in the manuscript.
